# Author Correction: The AUTOTAC chemical biology platform for targeted protein degradation via the autophagy-lysosome system

**DOI:** 10.1038/s41467-022-29845-w

**Published:** 2022-04-12

**Authors:** Chang Hoon Ji, Hee Yeon Kim, Min Ju Lee, Ah Jung Heo, Daniel Youngjae Park, Sungsu Lim, Seulgi Shin, Srinivasrao Ganipisetti, Woo Seung Yang, Chang An Jung, Kun Young Kim, Eun Hye Jeong, Sun Ho Park, Su Bin Kim, Su Jin Lee, Jeong Eun Na, Ji In Kang, Hyung Min Chi, Hyun Tae Kim, Yun Kyung Kim, Bo Yeon Kim, Yong Tae Kwon

**Affiliations:** 1 Cellular Degradation Biology Center and Department of Biomedical Sciences, College of Medicine, Seoul National University, Seoul, 03080 Korea; 2 AUTOTAC Bio Inc., Changkkyunggung-ro 254, Jongno-gu, Seoul, 03080 Korea; 3 Convergence Research Center for Brain Science, Brain Science Institute, Korea Institute of Science and Technology (KIST), Seoul, 02792 Korea; 4 Division of Bio-Medical Science & Technology, KIST School, University of Science and Technology (UST), Seoul, 02792 Korea; 5 Brown Cancer Center, University of Louisville, 529 S Jackson Street, Louisville, KY 40202 USA; 6 Anticancer Agents Research Center, Korea Research Institute of Bioscience and Biotechnology, Ochang, Cheongju, 28116 Korea; 7 Department of Chemisty, Pohang University of Science and Technology, Pohang, 37673 Korea; 8 Department of Biomolecular Science, KRIBB School, University of Science and Technology (UST), Daejeon, 34113 Korea; 9 SNU Dementia Research Center, College of Medicine, Seoul National University, Seoul, 110-799 Republic of Korea

**Keywords:** Protein quality control, Macroautophagy, Chemical tools, Lysosomes

Correction to: *Nature Communications* 10.1038/s41467-022-28520-4, published online 16 February 2022.

The original version of this Article omitted from the author list the 8^th^ author Srinivasrao Ganipisetti, who is from the University of Louisville. Consequently, Srinivasrao Ganipisetti’s initials were added to the Competing Interests section, which now reads as follows: ‘Seoul National University and AUTOTAC Bio, Inc. have filed patent applications (C.H.J., H.Y.K., M.J.L., A.J.H., S.G., J.E.N., H.T.K., and Y.T.K.; US 17/262,157 undergoing continuation-in-part, PCT/KR2019/009205 under examination; proof-of-concept AUTOTAC platform) based on the results of this study. The remaining authors declare no competing interests.‘ Additionally, Srinivasrao Ganipisetti’s initials were added to the Author Contributions, and the corrected sentence reads as follows: ‘ATLs binding the p62-ZZ domain were synthesized and modeled by S.G., K.Y.K., J.E.N., and H.T.K.’ This has been corrected in both the PDF and HTML versions of the Article.

