# Supplementary material for: The AUTOTAC chemical biology platform for targeted protein degradation via the autophagy-lysosome system
Source: Nat Commun. 2022 Feb 16;13:904. doi: 10.1038/s41467-022-28520-4 (PMC8850458; doi:10.1038/s41467-022-28520-4)
Supplement: Supplementary file 1 — Supplementary Information [file 41467_2022_28520_MOESM1_ESM.pdf]

# The AUTOTAC chemical biology platform for targeted protein degradation via the autophagy-lysosome system

**Chang Hoon Ji, Hee Yeon Kim, Min Ju Lee *et al.***

## SUPPLEMENTARY INFORMATION

## SUPPLEMENTARY METHODS

### Chemical synthesis and analytical data of Nt-Arg-mimicking compounds

$^{1}H$ NMR (Supplementary Fig. 8) and $^{13}C$ NMR (Supplementary Fig. 9) spectra were recorded on Bruker Avance III 500 MHz, 400 MHz and Bruker Fourier 300 MHz and TMS was used as an internal standard

LCMS (Supplementary Fig. 10) was taken on a quadrupole Mass Spectrometer on Agilent 1260HPLC and 6120MSD (Column: C18 (50 × 4.6 mm, 5 μm) operating in ES (+) or (-) ionization mode ; T = 30 °C; flow rate = 1.5 mL/min ; detected wavelength : 254 nm.

LC-HRMS (Supplementary Fig. 10) was taken by 3 methods. One was taken on Agilent 6550 iFunnel Q-TOF (Column : ZORBAX RRHD SB-C18 (80Å, 2.1x100mm 1.8μm)) operating in ES (+) or (-) ionization mode ; T = 25 °C ; flow rate = 0.5 mL/min ; detected wavelength : 254 nm. Another was taken on Agilent G6520 Q-TOF (Column: Agilent EC-C18 (4.6x50mm, 4.0um)) operating in ES (+) or (-) ionization mode ; T = 25 °C ; flow rate = 1.5 mL/min ; detected wavelength : 220nm and 254 nm. The other was taken on Agilent G6520 Q-TOF (Column: Xbridege C18 (4.6x50mm, 5.0um)) operating in ES (+) or (-) ionization mode ; T = 25 °C ; flow rate = 1.5 mL/min ; detected wavelength : 220nm and 254 nm.

**Scheme 1.** Synthesis of 2-((2-((3-((4-fluorobenzyl)oxy)benzyl)amino)ethyl)amino)ethan-1-ol (**yt-8-8**)

*1.1 Synthesis of 3-((4-fluorobenzyl)oxy)benzaldehyde (**1-1**)*

To a solution of 3-hydroxybenzaldehyde (**1**, 0.44 g, 3.60 mmol) in dimethylformamide was added $K_2CO_3$ (0.5 g, 3.60 mmol) and 1-(bromomethyl)-4-fluorobenzene (0.50 ml, 4.32 mmol) at r.t. The mixture was stirred at 60 °C for 4 hours. The reaction mixture was cooled to r.t and

extracted with ether and water. The organic layer was washed with brine, dried over $MgSO_4$, filtered and concentrated. The residue was purified by column chromatography on silica gel eluted with Ethyl acetate/Hexane (1:9) to afford 3-((4-fluorobenzyl)oxy)benzaldehyde (**1-1**, 0.70 g, 84 %).

$^1$H-NMR ($CDCl_3$, 400 MHz) δ (ppm) 9.98 (s, 1H), 7.48-7.40 (m, 5H), 7.25 (s, 1H), 7.11-7.02 (m, 2H), 5.08 (s, 2H) ; ESI-MS Calcd m/z for $C_{14}H_{11}FO_2$ $[M+H]^+$ 231.10 Found 231.07

*1.2 Synthesis of 2-((2-((3-((4-fluorobenzyl)oxy)benzyl)amino)ethyl)amino)ethan-1-ol (**yt-8-8**)*

To a solution of 3-((4-fluorobenzyl)oxy)benzaldehyde (**1-1**, 330 mg, 1.44 mmol) in methanol (4 ml) was added 2-(2-aminoethylamino)ethanol (0.16 ml, 1.1 mmol) at r.t. The reaction mixture was stirred at 65 °C overnight. After checking TLC for monitoring imine formation, the mixture was cooled to 0 °C. Then to the mixture was added $NaBH_4$ (109 mg, 2.88 mmol) and stirred at r.t for 1 hour. The mixture was concentrated, diluted with water and extracted with Ethyl Acetate. The organic layer was dried over $MgSO_4$, filtered and concentrated. The residue was purified by column chromatography on silica gel to afford 2-((2-((3-((4-fluorobenzyl)oxy)benzyl)amino)ethyl)amino)ethan-1-ol (**yt-8-8**, 162 mg, 35 %) as a white powder.

$^1$H-NMR (DMSO_$d_6$, 500 MHz) δ (ppm) 7.54 – 7.42 (m, 2H), 7.28 – 7.14 (m, 3H), 6.99 (dd, J = 2.1, 1.6 Hz, 1H), 6.86 (ddd, J = 11.9, 8.2, 4.7 Hz, 2H), 5.06 (s, 2H), 3.65 (s, 2H), 3.42 (t, J = 5.7 Hz, 2H), 2.60 – 2.53 (m, 6H) ; $^{13}$C-NMR (DMSO_$d_6$, 125 MHz) δ (ppm) 162.72, 160.78, 158.27, 142.89, 133.48, 129.98, 129.12, 120.39, 115.33, 115.16, 114.18, 112.78, 68.33, 60.47, 52.89, 51.73, 48.99, 48.44 ; HRMS Calcd m/z for $C_{18}H_{23}FN_2O_2$ $[M+H]^+$ 319.1816 Found 319.1816

**Scheme 2.** Synthesis of (*R*)-1-(3,4-diphenethoxyphenoxy)-3-(isopropylamino)propan-2-ol (**yok-2204**)

*2.1 Synthesis of 3,4-diphenethoxybenzaldehyde (**2-1**)*

To a solution of 3,4-dihydroxybenzaldehyde (**2**, 0.50 g, 3.62 mmol) in dimethylformamide was added $K_2CO_3$ (1.50 g, 10.86 mmol) and (bromoethyl)benzene (1.09 ml, 7.96 mmol) at r.t. The mixture was stirred at 60 °C for 4 hours. The reaction mixture was cooled to r.t. The reaction mixture was extracted with $H_2O$ and diethyl ether. The organic layer was washed with brine, dried over $Na_2SO_4$ and concentrated. The crude was purified by column chromatography (Hexane/Ethyl acetate, 9:1) to afford 3,4-diphenethoxybenzaldehyde (**2-1**, 1.10 g, 88 %).

$^1$H-NMR ($CDCl_3$, 300 MHz) δ (ppm) 9.84 (s, 1H), 7.43-7.26 (m, 7H), 6.96 (d, J = 9.0 Hz, 1H), 5.62 (s, 1H), 4.36 (t, J = 6.0 Hz, 2H), 3.17 (t, J = 6.0 Hz, 2H); ESI-MS Calcd m/z for C23H22O3 $[M+H]^+$ 347.33 Found 347.16

*2.2 Synthesis of 3,4-diphenethoxyphenol (**2-2**)*

To a solution of 3,4-diphenethoxybenzaldehyde (**2-1**, 1.04 g, 3.0 mmol) in dichloromethane (15 mL) was added meta-chloroperoxybenzoic acid (0.78g, 4.5 mmol) in portions. The mixture was stirred at r.t for 4 hours. Then the mixture was diluted with ethyl acetate, washed with saturated aq. $Na_2CO_3$ solution. The organic layer was dried over $Na_2SO_4$, filtered and concentrated. The mixture was dissolved in methanol (10 mL) and added 6N aq. NaOH solution. The mixture was stirred at r.t for 30 min. Then The mixture was added aq. 4N HCl solution and stirred at r.t for 30 min. Then The mixture was diluted with ethyl acetate, washed with brine, dried over anhydrous $Na_2SO_4$ and filtered. The residue was concentrated and purified by column chromatography (Hexane/Ethyl acetate, 7:3) to afford 3,4-diphenethoxyphenol (**2-2**, 0.89 g, 89 %).

$^1$H-NMR ($CDCl_3$, 300 MHz) δ (ppm) 7.35-7.22 (m, 10H), 6.76 (d, J = 9.0 Hz, 1H), 6.44 (d, J = 3.0 Hz, 1H), 6.30 (dd, J = 3.0, 6.0 Hz, 1H), 4.74 (s, 1H), 4.13 (td, J = 3.0, 9.0 Hz, 4H), 3.10 (td, J = 3.0, 9.0 Hz, 4H)

*2.3 Synthesis of (R)-2-((3,4-diphenethoxyphenoxy)methyl)oxirane (**2-3**)*

To a solution of 3,4-diphenethoxyphenol (**2-2**, 334 mg, 1.0 mmol) in ethanol (10 mL) was added KOH (66 mg, 1.2 mmol) and $H_2O$ (1 mL). The mixture was added (*R*)-2-(chloromethyl)oxirane (41 μL, 5 mmol). The mixture was stirred at r.t for 5 hours. The mixture was concentrated, diluted with ethyl acetate and washed with water and brine. Then the organic layer was dried over anhydrous $Na_2SO_4$ and filtered. The residue was concentrated and purified by using column chromatography (Hexane/Ethyl acetate, 9:1) to give (*R*)-2-((3,4-diphenethoxyphenoxy)methyl)oxirane (**2-3**, 312 mg, 80 %).

$^1$H-NMR ($CDCl_3$, 500 MHz) δ (ppm) 7.31-7.20 (m, 10H), 6.77 (d, J = 9 Hz, 1H), 6.52 (d, J = 3 Hz, 1H), 6.37 (dd, J = 9, 3 Hz, 1H), 4.17-4.09 (m, 5H), 3.85 (dd, J = 11, 5.5 Hz, 1H), 3.31-3.29 (m, 1H), 3.13-3.06 (m, 4H), 2.87 (t, J = 5 Hz, 1H), 2.71 (dd, J = 5, 2.5 Hz, 1H)

*2.4 Synthesis of (R)-1-(3,4-diphenethoxyphenoxy)-3-(isopropylamino)propan-2-ol (**yok-2204**)*

The mixture of (*R*)-2-((3,4-diphenethoxyphenoxy)methyl)oxirane (**2-3**, 10.4 mg, 25 nmol) and propan-2-amine (10 μL, 125 nmol) in ethanol (1 mL) was stirred at r.t for 4 hours. The reaction mixture was concentrated, diluted with dichloromethane and extracted with dichloromethane and water. Then the organic layer was washed with brine, dried over $Na_2SO_4$ and filtered. The residue was concentrated and purified by column chromatography (dichloromethane/dichloromethane, 19:1) to give (*R*)-1-(3,4-diphenethoxyphenoxy)-3-(isopropylamino)propan-2-ol (**yok-2204**, 10 mg, 88 %).

$^{1}H$-NMR ($DMSO\_d_{6}$, 500 MHz) δ (ppm) 7.37 – 7.25 (m, 8H), 7.21 (ddd, J = 7.2, 3.7, 2.1 Hz, 2H), 6.82 (d, J = 8.8 Hz, 1H), 6.56 (d, J = 2.8 Hz, 1H), 6.39 (dd, J = 8.8, 2.8 Hz, 1H), 4.91 (brs, 1H), 4.13 (t, J = 6.7 Hz, 2H), 4.03 (t, J = 6.8 Hz, 2H), 3.86 (q, J = 7.2 Hz, 1H), 3.77 (q, J = 5.5 Hz, 2H), 3.00 (t, J = 6.7 Hz, 2H), 2.95 (t, J = 6.8 Hz, 2H), 2.66 (ddd, J = 15.6, 12.1, 5.3 Hz, 2H), 2.52 (d, J = 6.4 Hz, 1H), 0.96 (dd, J = 6.2, 2.8 Hz, 6H) ; $^{13}C$-NMR ($DMSO\_d_{6}$, 125 MHz) δ (ppm) 153.69, 149.46, 142.19, 138.64, 138.53, 129.03, 129.00, 128.22, 126.22, 126.16, 115.78, 105.06, 102.16, 71.22, 70.19, 68.94, 68.45, 50.10, 48.22, 22.98, 22.96 ; HRMS Calcd m/z for $C_{28}H_{35}NO_{4}$ $[M+H]^{+}$ 450.2639 Found 450.2639

**Scheme 3.** Synthesis of (*R*)-1-(4-(benzyloxy)-3-phenethoxyphenoxy)-3-(isopropylamino)propan-2-ol (**yok-1304**)

*3.1 Synthesis of 4-(benzyloxy)-3-hydroxybenzaldehyde (**3-1**)*

To a solution of 3,4-dihydroxybenzaldehyde (**2**, 20.0 g, 145 mmol) and (bromomethyl)benzene (24.8 g, 145 mmol) in acetonitrile (400 mL) was add $NaHCO_{3}$ (14.6 g, 174 mmol) at 25 °C. The mixture was stirred overnight at 80 °C. The reaction was concentrated. The residue was quenched with 1N HCl and extracted with ethyl acetate. The organic layer was washed with brine, dried over $Na_{2}SO_{4}$, filtered and concentrated. The residue was purified by column chromatography on silica gel, eluted with ethyl acetate/ petroleum ether (1:20~1:10) to afford 4-(benzyloxy)-3-hydroxybenzaldehyde (**3-1**, 10.0 g, 30 %) as a white solid.

$^{1}H$-NMR ($DMSO\_d_{6}$, 400 MHz) δ (ppm) 9.76 (s, 1H), 9.66 (s, 1H), 7.49-7.48 (m, 2H), 7.42-7.34 (m, 4H), 7.29 (d, J = 2.0 Hz, 1H), 7.20 (d, J = 8.4 Hz, 1H), 5.23 (s, 2H) ; ESI-MS Calcd m/z for $C_{14}H_{12}O_{3}$ $[M+H]^{+}$ 229.20 Found 229.08

*3.2 Synthesis of 4-(benzyloxy)-3-phenethoxybenzaldehyde (**3-2**)*

To a solution of 4-(benzyloxy)-3-hydroxybenzaldehyde (**3-1**, 10.0 g, 43.9 mmol) and (2-bromoethyl)benzene (9.71 g, 52.6 mmol) in dimethylformamide (100 mL) was added $Cs_2CO_3$ (43.0 g, 132 mmol). The mixture was stirred at 80 $^oC$ overnight. The mixture was added water and extracted with ethyl acetate. The organic layer was washed with brine, dried over $Na_2SO_4$, filtered and concentrated. The residue was purified by column chromatography on silica gel, eluted with ethyl acetate/ petroleum ether (20:1~10:1) to afford 4-(benzyloxy)-3-phenethoxybenzaldehyde (**3-2**, 3.7 g, 25 %).

$^1$H-NMR ($CDCl_3$, 400 MHz) δ (ppm) 9.83 (s, 1H), 7.46-7.25 (m, 12H), 7.02 (d, J = 7.6 Hz, 1H), 5.22 (s, 2H), 4.31 (t, J = 6.8 Hz, 2H), 3.18 (t, J = 6.8 Hz, 2H) ; ESI-MS Calcd m/z for $C_{22}H_{20}O_3$ $[M+H]^+$ 333.20 Found 333.14

*3.3 Synthesis of 4-(benzyloxy)-3-phenethoxyphenol (**3-3**)*

To a solution of 4-(benzyloxy)-3-phenethoxybenzaldehyde (**3-2**, 3.70 g, 11.1 mmol) in dichloromethane (40 mL) was added meta-chloroperoxybenzoic acid (2.90 g, 16.7 mmol) in portions. The mixture was stirred at r.t for 2 hours. The mixture was washed with saturated $NaHCO_3$ solution, and concentrated. The mixture was dissolved in methanol (25 mL) and added 5N KOH (2.5 mL, 12.3 mmol). The mixture was stirred at r.t for 1 hour. The mixture was added ice water and filtered. The solid was concentrated to afford 4-(benzyloxy)-3-phenethoxyphenol (**3-3**, 3.4 g, 96 %).

$^1$H-NMR (DMSO_$d_6$, 400 MHz) δ (ppm) 9.01 (s, 1H), 7.37-7.21 (m, 10H), 6.80 (d, J = 8.8 Hz, 1H), 6.43 (s, 1H), 6.22 (dd, J = 2.4, 8.4 Hz, 1H), 4.87 (s, 2H), 4.14 (t, J = 6.8 Hz, 2H), 3.03 (t, J = 6.4 Hz, 2H) ; ESI-MS Calcd m/z for $C_{21}H_{20}O_3$ $[M+H]^+$ 321.80 Found 321.14

*3.4 Synthesis of (R)-2-((4-(benzyloxy)-3-phenethoxyphenoxy)methyl)oxirane (**3-4**)*

To a solution of 4-(benzyloxy)-3-phenethoxyphenol (**3-3**, 3.4 g, 10.6 mmol) in ethanol (50 mL) was added KOH (0.7 g, 12.8 mmol) and $H_2O$ (5 mL). The mixture was added (*R*)-2-(chloromethyl)oxirane (2.9 g, 31.9 mmol). The mixture was stirred at 30 $^{o}$C overnight. The mixture was added water and filtered. The solid was concentrated to give (*R*)-2-((4-(benzyloxy)-3-phenethoxyphenoxy)methyl)oxirane (**3-4**, 3.6 g, 90 %).

$^{1}$H-NMR (DMSO_d$_6$, 400 MHz) δ (ppm) 7.41-7.19 (m, 10H), 6.90 (d, J = 8.8 Hz, 1H), 6.65 (d, J = 2.8 Hz, 1H), 6.42 (dd, J = 2.4, 8.8 Hz, 1H), 4.93 (s, 2H), 4.26-4.18 (m, 3H), 3.77-3.72 (m, 1H), 3.30-3.27 (m, 1H), 3.04 (t, J = 6.4 Hz, 2H), 2.82 (t, J = 4.8 Hz, 1H), 2.69-2.67 (m, 1H) ; ESI-MS Calcd m/z for $C_{24}H_{24}O_4$ $[M+H]^+$ 377.10 Found 377.17

*3.5 Synthesis of (R)-1-(4-(benzyloxy)-3-phenethoxyphenoxy)-3-(isopropylamino)propan-2-ol **(yok-1304)***

The mixture of (*R*)-2-((4-(benzyloxy)-3-phenethoxyphenoxy)methyl)oxirane (**3-4**, 3.6 g, 9.6 mmol) and propan-2-amine (2.8 g, 47.9 mmol) in methanol (100 mL) was stirred overnight at 50 $^{o}$C. The reaction mixture was concentrated and purified by chromatography (dichloromethane/methanol=15/1) to give (*R*)-1-(4-(benzyloxy)-3-phenethoxyphenoxy)-3-(isopropylamino)propan-2-ol (**yok-1304**, 1.0 g, 24 %).

$^{1}$H-NMR (DMSO_d$_6$, 500 MHz) δ (ppm) 7.37 – 7.25 (m, 9H), 7.21 (ddd, J = 7.2, 3.7, 2.1 Hz, 1H), 6.82 (d, J = 8.8 Hz, 1H), 6.56 (d, J = 2.8 Hz, 1H), 6.39 (dd, J = 8.8, 2.8 Hz, 1H), 4.91 (s, 2H), 4.13 (t, J = 6.7 Hz, 2H), 4.03 (t, J = 6.8 Hz, 1H), 3.86 (q, J = 7.2 Hz, 2H), 3.77 (q, J = 5.5 Hz, 2H), 3.00 (t, J = 6.7 Hz, 2H), 2.66 (m, 2H), 2.52 (dd, J = 10, 5 Hz, 1H), 0.96 (dd, J = 6.2, 2.8 Hz, 6H) ; $^{13}$C-NMR (DMSO_d$_6$, 125 MHz) δ (ppm) 153.85, 149.66, 141.88, 138.56, 137.67, 129.07, 128.25, 128.20, 127.61, 127.49, 126.22, 116.28, 104.88, 101.91, 71.17,

71.08, 68.80, 68.30, 49.93, 48.29, 35.04, 22.75 ; HRMS Calcd m/z for $C_{27}H_{33}NO_4$ $[M+H]^+$ 436.2482 Found 436.2482

**Scheme 4.** Synthesis of 2-((3-((4-fluorobenzyl)oxy)benzyl)amino)ethan-1-ol (**YTK-105**)

*4.1 Synthesis of 2-((3-((4-fluorobenzyl)oxy)benzyl)amino)ethan-1-ol (**YTK-105**)*

To a solution of 3-((4-fluorobenzyl)oxy)benzaldehyde (**1-1**, 2.00 g, 8.70 mmol) and 2-aminoethanol (530 mg, 8.70 mmol) in methanol (30 mL) was stirred for 1 hour at r.t. The mixture was added $NaBH(OAc)_3$ (5.53 g, 26.1 mmol) at r.t, then stirred for 1 hour at r.t. The reaction was quenched with saturated aq. $NH_4Cl$ solution, extracted with ethyl acetate and concentrated. The crude was purified by column chromatography (dichloromethane/methanol=20/1~10/1) to give 2-((3-((4-fluorobenzyl)oxy)benzyl)amino)ethan-1-ol (**YTK-105**, 1.10g, 46 %) as grey solid.

$^1$H-NMR (DMSO_$d_6$, 500 MHz) δ (ppm) 7.55 – 7.42 (m, 2H), 7.27 – 7.16 (m, 3H), 6.99 (d, J = 1.9 Hz, 1H), 6.89 (d, J = 7.5 Hz, 1H), 6.85 (dd, J = 7.9, 2.3 Hz, 1H), 5.08 (d, J = 15.1 Hz, 2H), 4.47 (brs, 1H), 3.67 (s, 2H), 3.45 (t, J = 5.8 Hz, 2H), 2.54 (t, J = 5.8 Hz, 2H), 2.14 (brs, 1H) ; $^{13}$C-NMR (DMSO_$d_6$, 125 MHz) δ (ppm) 162.70, 160.77, 158.27, 142.77, 133.45, 129.95, 129.89, 129.11, 120.39, 115.31, 114.23, 112.78, 68.33, 60.41, 52.80, 51.02 ; HRMS Calcd m/z for $C_{16}H_{18}FNO_2$ $[M+H]^+$ 276.1395 Found 276.1394

**Scheme 5.** Synthesis of *N*-(1-(3,4-bis(benzyloxy)phenyl)-5,8,11-trioxa-2-azatridecan-13-yl)-4-phenylbutanamide (**PBA-1105**)

*5.1 Synthesis of 3,4-bis(benzyloxy)benzaldehyde (**5-1**)*

To a solution of 3,4-dihydroxybenzaldehyde (**2**, 0.50 g, 3.62 mmol) in dimethylformamide was added $K_2CO_3$ (1.50 g, 10.86 mmol) and (bromomethyl)benzene (0.92 mL, 7.96 mmol) at r.t. The mixture was stirred at 60 °C for 4 hours. The reaction mixture was cooled to r.t and extracted with ether and water. The organic layer was washed with brine, dried over $MgSO_4$, filtered and concentrated. The residue was purified by column chromatography on silica gel to afford 3,4-bis(benzyloxy)benzaldehyde (**5-1**, 1.04 g, 90 %).

$^1$H-NMR ($CDCl_3$, 300 MHz) δ (ppm) 9.81 (s, 1H), 7.49-7.31 (m, 12H), 7.04 (d, J = 8.3 Hz, 1H), 5.27 (s, 2H), 5.22 (s, 2H) ; ESI-MS Calcd m/z for $C_{21}H_{18}O_3$ $[M+H]^+$ 319.33 Found 319.13

*5.2 Synthesis of N-(2-(2-(2-(2-aminoethoxy)ethoxy)ethoxy)ethyl)-4-phenylbutanamide (**5-2**)*

To a solution of 2,2'-((oxybis(ethane-2,1-diyl))bis(oxy))bis(ethan-1-amine) (21 g, 73.1 mmol) in dichloromethane (400 mL) was added Hydroxybenzotriazole (HOBT, 12.3 g, 91.5 mmol), 1-ethyl-3-(3-dimethylaminopropyl)carbodiimide (EDCI, 17.6 g, 91.5 mmol) and trimethylamine (18.5 g, 180 mmol). The solution of 4-phenylbutanoic acid (**5**, 15 g, 91.5 mmol) in dichloromethane (200 mL) was added at 0 $^0$C. The mixture was stirred at r.t overnight. The above solution was poured into water and extracted with dichloromethane. The organic layer was washed with brine, dried over $Na_2SO_4$ and concentrated. The crude was purified by column chromatography on silica gel (dichloromethane/methanol=15:1) to give *N*-(2-(2-(2-(2-aminoethoxy)ethoxy)ethoxy)ethyl)-4-phenylbutanamide (**5-2**, 12 g, 49 %) as yellow oil.

$^1$H-NMR ($CDCl_3$, 400 MHz) δ (ppm) 7.29-7.26 (m, 2H), 7.19-7.17 (m, 3H), 3.65-3.60 (m, 8H), 3.57-3.52 (m, 4H), 3.46 (q, J = 4.8 Hz, 3H), 2.86 (t, J = 5.2 Hz, 2H), 2.65 (t, J = 7.6 Hz, 2H), 2.44 (s, 4H), 2.22 (t, J = 7.2 Hz, 2H), 1.99-1.95 (m, 2H) ; ESI-MS Calcd m/z for $C_{18}H_{30}N_2O_4$ $[M+H]^+$ 339.10 Found 339.22.

*5.3 Synthesis of N-(1-(3,4-bis(benzyloxy)phenyl)-5,8,11-trioxa-2-azatridecan-13-yl)-4-*

*phenylbutanamide (**PBA-1105**)*

To a solution of *N*-(2-(2-(2-(2-aminoethoxy)ethoxy)ethoxy)ethyl)-4-phenylbutanamide (**5-2**, 10 g, 29.6 mmol) in methanol (150 mL) was added 3,4-bis(benzyloxy)benzaldehyde (**5-1**, 10.3 g, 32.3 mmol). The mixture was stirred for 5 hours at 65 $^{0}$C. $NaBH_4$ (2.2 g, 57.9 mmol) was added at r.t. The reaction mixture was stirred for 5 hours at r.t. The reaction mixture was poured into water. The solution was extracted with ethyl acetate. The combined organic layer was washed with brine, dried over $Na_2SO_4$ and concentrated. The crude was purified by column chromatography on silica gel (dichloromethane/methanol=15:1) to give *N*-(1-(3,4-bis(benzyloxy)phenyl)-5,8,11-trioxa-2-azatridecan-13-yl)-4-phenylbutanamide (**PBA-1105**, 10.3 g, 54 %) as yellow oil.

$^{1}$H-NMR (DMSO_$d_6$, 500 MHz) δ (ppm) 7.88 (t, J = 5.6 Hz, 1H), 7.49 – 7.41 (m, 4H), 7.41 – 7.34 (m, 4H), 7.34 – 7.23 (m, 4H), 7.20 – 7.12 (m, 3H), 7.07 (d, J = 1.9 Hz, 1H), 6.97 (d, J = 8.2 Hz, 1H), 6.82 (dd, J = 8.2, 1.9 Hz, 1H), 5.10 (d, J = 4.1 Hz, 4H), 3.62 (s, 2H), 3.52 – 3.41 (m, 10H), 3.38 (t, J = 5.9 Hz, 2H), 3.19 (q, J = 5.8 Hz, 2H), 2.59 (t, J = 5.7 Hz, 2H), 2.56 – 2.51 (m, 2H), 2.08 (t, J = 7.4 Hz, 2H), 1.82 – 1.71 (m, 2H) ; $^{13}$C-NMR (DMSO_$d_{6,}$ 125 MHz) 171.89, 148.17, 147.06, 141.80, 137.46, 137.36, 133.54, 128.35, 128.32, 128.25, 127.73, 127.69, 127.57, 127.47, 125.73, 120.64, 114.48, 114.36, 70.19, 70.09, 69.83, 69.77, 69.75, 69.62, 69.56, 69.16, 52.41, 47.75, 38.46, 34.76, 34.66., 27.10 ; HRMS Calcd m/z for $C_{39}H_{48}N_2O_6$ $[M+H]^+$ 641.3584 Found 641.3585.

**Scheme 6.** Synthesis of 3-(3-(benzo[*d*][1,3]dioxol-5-yl)-1*H*-pyrazol-5-yl)-*N*-(2-(2-(2-((3-((4-fluorobenzyl)oxy)benzyl)amino)ethoxy)ethoxy)ethyl)aniline (**Anle138b-F105**)

*6.1 Synthesis of 1-(benzo[d][1,3]dioxol-5-yl)-3-(3-bromophenyl)propane-1,3-dione (**6-1**)*

To a solution of 1-(benzo[d][1,3]dioxol-5-yl)ethan-1-one (**6**, 20.0 g, 122 mmol) and NaH (6.10 g, 152 mmol, 60% in mineral oil) in dry dimethylsulfoxide (120 mL) was stirred at 15 $^{o}$C for 30 min. Then a solution of methyl 3-bromobenzoate (32.8 g, 152 mmol) in dimethylsulfoxide (60 mL) was added at 20 $^{o}$C. The resulting mixture was stirred at 25 $^{o}$C for 2 hours. The reaction was quenched with saturated aq. $NH_4Cl$ solution. The mixture was poured into water and petroleum ether and stirred for 30 min at r.t. Then filtered to give 1-(benzo[*d*][1,3]dioxol-5-yl)-3-(3-bromophenyl)propane-1,3-dione (**6-1**, 35.0 g, crude) as yellow solid.

$^{1}$H-NMR ($CDCl_3$, 400 MHz) δ (ppm) 8.07 (s, 1H), 7.88 (d, J = 7.6 Hz, 1H), 7.69-7.59 (m, 2H), 7.46 (s, 1H), 7.37-7.30 (m, 1H), 6.90 (d, J = 8 Hz, 1H), 6.69 (s, 1H), 6.07 (s, 2H) ; ESI-MS Calcd m/z for $C_{16}H_{11}BrO_4$ $[M+H]^+$ 347.00 Found 346.98

*6.2 Synthesis of 3-(benzo[d][1,3]dioxol-5-yl)-5-(3-bromophenyl)-1H-pyrazole (**6-2**)*

To a solution of 1-(benzo[*d*][1,3]dioxol-5-yl)-3-(3-bromophenyl)propane-1,3-dione (**6-1**, 35.0 g, 101 mmol) and 98% $N_2H_4.H_2O$ (5.80 g, 116 mmol) in ethanol (600 mL) was refluxed for 2 hours. The mixture was cooled to r.t and filtered to give 3-(benzo[*d*][1,3]dioxol-5-yl)-5-(3-bromophenyl)-1*H*-pyrazole (**6-2**, 25.0 g, 63 %) as off-white solid.

$^{1}$H-NMR (DMSO_d$_6$, 400 MHz) δ (ppm) 13.08 (s, 1H), 8.02 (s, 1H), 7.82 (d, J = 7.6 Hz, 1H), 7.52-7.32 (m, 4H), 7.08 (s, 1H), 6.98 (d, J = 8 Hz, 1H), 6.05 (brs, 2H) ; ESI-MS Calcd m/z for $C_{16}H_{11}BrN_2O_2$ $[M+H]^+$ 344.00 Found 343.00

*6.3 Synthesis of N-(2-(2-(2-aminoethoxy)ethoxy)ethyl)-3-(3-(benzo[d][1,3]dioxol-5-yl)-1H-pyrazol-5-yl)aniline (**6-3**)*

To a solution of 3-(benzo[*d*][1,3]dioxol-5-yl)-5-(3-bromophenyl)-1*H*-pyrazole (**6-2**, 25.0 g,

72.9 mmol) and 2,2'-(ethane-1,2-diylbis(oxy))diethanamine (32.4 g, 219 mmol) in 1,4-dioxane (250 mL) was added NaH (8.75 g, 219 mmol, 60% in mineral oil) slowly at 25 $^oC$. Then added $Pd_2(dba)_3$ (2.5 g, 2.73 mmol) and 2,2′-bis(diphenylphosphino)-1,1′-binaphthyl (BINAP, 5 g, 8.05 mmol) at 25 $^oC$ under $N_2$. The mixture was stirred overnight at 100 $^oC$ under $N_2$. The reaction was cooled to r.t, then quenched with water, extracted with ethyl acetate and concentrated. The crude was purified by column chromatography (dichloromethane/methanol=50/1~5/1) to give the *N*-(2-(2-(2-aminoethoxy)ethoxy)ethyl)-3-(3-(benzo[*d*][1,3]dioxol-5-yl)-1*H*-pyrazol-5-yl)aniline (**6-3**, 12.0 g, 40 %).

$^1H$-NMR ($CDCl_3$, 400 MHz) δ (ppm) 7.29 (s, 2H), 7.22-7.20 (m, 1H), 7.01-6.99 (m, 2H), 6.83 (d, J = 8 Hz, 1H), 6.69 (s, 1H), 6.58 (d, J = 8 Hz, 1H), 5.97 (s, 1H), 3.73-3.71 (m, 2H), 3.66-3.55 (m, 6H), 3.47 (s, 2H), 3.29-3.27 (m, 2H), 3.01-2.98 (m, 2H) ; ESI-MS Calcd m/z for $C_{22}H_{26}N_4O_4$ $[M+H]^+$ 411.20 Found 410.20

*6.4 Synthesis of 3-(3-(benzo[d][1,3]dioxol-5-yl)-1H-pyrazol-5-yl)-N-(2-(2-(2-((3-((4-fluorobenzyl)oxy)benzyl)amino)ethoxy)ethoxy)ethyl)aniline (**Anle138b-F105**)*

To a solution of *N*-(2-(2-(2-aminoethoxy)ethoxy)ethyl)-3-(3-(benzo[*d*][1,3]dioxol-5-yl)-1*H*-pyrazol-5-yl)aniline (**6-3**, 12.0 g, 29.3 mmol) and 3-((4-fluorobenzyl)oxy)benzaldehyde (**1-1**, 6.73 g, 29.3 mmol) in methanol (120 mL) was stirred for 1 hour at r.t. The mixture was added $NaBH(OAc)_3$ (18.6 g, 87.8 mmol) at r.t, then stirred for 1 hour at r.t. The reaction was quenched with saturated aq. $NH_4Cl$ solution, extracted with ethyl acetate and concentrated. Another 3 g batch was carried out as the above procedure. The crude was purified by prep-HPLC to give 3-(3-(benzo[*d*][1,3]dioxol-5-yl)-1*H*-pyrazol-5-yl)-*N*-(2-(2-(2-((3-((4-fluorobenzyl)oxy)benzyl)amino)ethoxy)ethoxy)ethyl)aniline (**Anle138b-F105**, 10.0g, 44 %) as yellow oil.

$^{1}$H-NMR (DMSO_$d_6$, 500 MHz) δ (ppm) 13.14 (s, 1H), 7.51 – 7.44 (m, 2H), 7.39 (d, J = 1.7 Hz, 1H), 7.34 (d, J = 7.8 Hz, 1H), 7.26 – 7.16 (m, 3H), 7.11 (t, J = 7.8 Hz, 1H), 7.05 – 7.00 (m, 2H), 7.00 – 6.94 (m, 3H), 6.91 (d, J = 7.6 Hz, 1H), 6.88 – 6.83 (m, 1H), 6.57 (d, J = 7.5 Hz, 1H), 6.05 (s, 2H), 5.64 (brs, 1H), 5.04 (s, 2H), 3.72 (s, 2H), 3.61 – 3.48 (m, 9H), 3.24 (q, J = 5.6 Hz, 2H), 2.67 (t, J = 5.7 Hz, 2H) ; $^{13}$C-NMR (DMSO_$d_6$, 125 MHz) δ (ppm) 162.71, 160.77, 158.26, 149.04, 147.72, 133.38, 133.36, 129.95, 129.89, 129.24, 120.68, 118.75, 115.31, 115.14, 114.55, 113.13, 108.58, 105.55, 101.09, 99.07, 69.69, 69.68, 69.52, 69.06, 68.35, 54.95, 52.41, 47.68, 42.67 ; HRMS Calcd m/z for $C_{36}H_{37}FN_4O_5$ $[M+H]^+$ 625.2820 Found 625.2821.

**Scheme 7.** Synthesis of (*R*)-1-(4-(benzyloxy)-3-(3-phenylpropoxy)phenoxy)-3-((2-(2-(2-(4-(2-phenyl-5,7-bis(trifluoromethyl)pyrazolo[1,5-*a*]pyrimidin-3-yl)phenoxy)ethoxy)ethoxy)ethyl)amino)propan-2-ol (**PHTPP-1304**)

*7.1 Synthesis of 4-(benzyloxy)-3-(3-phenylpropoxy)benzaldehyde (**7-1**)*

To a solution of 4-(benzyloxy)-3-hydroxybenzaldehyde (**3-1**, 120 g, 526 mmol) in tetrahydrofuran (2 L) were added 2-phenylethanol (85.9 g, 631 mmol), $PPh_3$ (206.9 g, 789 mmol) and diisopropyl azodicarboxylate (DIAD, 159.5 g, 789 mmol). The mixture was stirred at 65 $^{o}$C for 16 hours. The reaction was concentrated and purification by silica gel, eluted with ethyl acetate/petroleum ehter (1:15~1:10) to afford 4-(benzyloxy)-3-(3-phenylpropoxy)benzaldehyde (**7-1**, 100 g, 55 %) as a white solid.

$^{1}$H-NMR (DMSO_$d_6$, 400 MHz) δ (ppm) 9.80 (s, 1H), 7.55-7.48 (m, 3H), 7.42-7.38 (m, 3H), 7.37 (dd, J = 10.8, 5.6 Hz, 1H), 7.29-7.25 (m, 3H), 7.21-7.18 (m, 3H), 5.27 (s, 2H), 4.05 (t, J = 6.4 Hz, 2H), 2.76 (t, J = 7.2 Hz, 2H), 2.06-2.02 (m, 2H) ; ESI-MS Calcd m/z for $C_{23}H_{22}O_3$

$[M+H]^+$ 347.80 Found 347.16

*7.2 Synthesis of 4-(benzyloxy)-3-(3-phenylpropoxy)phenol (7-2)*

To a solution of 4-(benzyloxy)-3-(3-phenylpropoxy)benzaldehyde (**7-1**, 100 g, 289 mmol) in dichloromethane (900 mL) was added meta-chloroperoxybenzoic acid (74.7 g, 433 mmol). The mixture was stirred at r.t for 16 hours. Then the reaction was filtered. The filtration was concentrated. The residue was purified by silica gel, eluted with ethyl acetate/petroleum ehter (1:15~1:5) to afford 4-(benzyloxy)-3-(3-phenylpropoxy)phenol (**7-2**, 66 g, 68 %) as an off-white solid.

$^1$H-NMR (DMSO_d$_6$, 400 MHz) δ (ppm) 9.00 (s, 1H), 7.43 (d, J = 7.2 Hz, 2H), 7.38-7.26 (m, 5H), 7.21-7.18 (m, 3H), 6.81 (d, J = 8.8 Hz, 1H), 6.39 (d, J = 2.4 Hz, 1H), 6.22 (dd, J = 2.8, 8.4 Hz, 1H), 4.96 (s, 2H), 3.90 (t, J = 6 Hz, 2H), 2.74 (t, J = 7.2 Hz, 2H), 2.02-1.98 (m, 2H) ; ESI-MS Calcd m/z for $C_{22}H_{22}O_3$ $[M+H]^+$ 334.80 Found 335.16

*7.3 Synthesis of (R)-2-((4-(benzyloxy)-3-(3-phenylpropoxy)phenoxy)methyl)oxirane (7-3)*

To a solution of 4-(benzyloxy)-3-(3-phenylpropoxy)phenol (**7-2**, 40 g, 120 mmol) in ethanol (800 mL) were added water (40 mL) and KOH (8.0 g, 143 mmol). Then (R)-2-(chloromethyl)oxirane (33.2 g, 359 mmol) was added to the reaction. The resulting mixture was stirred at r.t for 16 hours. Then the reaction was quenched by addition water, extracted with ethyl acetate. The organic layer was washed with brine, dried over $Na_2SO_4$, filtered and concentrated. The residue was purified by silica gel, eluted with ethyl acetate/petroleum ehter (1:15~1:10) to afford (*R*)-2-((4-(benzyloxy)-3-(3-phenylpropoxy)phenoxy)methyl)oxirane (**7-3**, 30 g, 64 %) as a white solid.

$^1$H-NMR (DMSO_d$_6$, 400 MHz) δ (ppm) 7.43 (d, J = 7.2 Hz, 2H), 7.38-7.26 (m, 5H), 7.21-7.16 (m, 3H), 6.91 (d, J = 8.8 Hz, 1H), 6.61 (d, J = 3.2 Hz, 1H), 6.42 (dd, J = 2.8, 8.4 Hz, 1H),

5.02 (s, 2H), 4.23 (dd, J = 2.8, 11.2 Hz, 1H), 3.97 (t, J = 6 Hz, 2H), 3.74 (dd, J = 6.4, 11.2 Hz, 1H), 3.28 (m, 1H), 2.82 (t, J = 4.8 Hz, 1H), 2.75 (t, J = 7.2 Hz, 2H), 2.68 (dd, J = 2.4, 4.8 Hz, 1H), 2.03-1.99 (m, 2H) ; ESI-MS Calcd m/z for $C_{25}H_{26}O_4$ $[M+H]^+$ 391.90 Found 391.18

*7.4 Synthesis of tert-butyl (2-(2-(2-hydroxyethoxy)ethoxy)ethyl)carbamate (**7-4**)*

To a solution of 2-(2-(2-aminoethoxy)ethoxy)ethan-1-ol (**7**, 50 g, 336 mmol) in dichloromethane (500 mL) were added triethylamine (TEA, 40.4 g, 400 mmol) and $(Boc)_2O$ (80.6 g, 370 mmol). The mixture was stirred at r.t for 16 hours. The reaction was concentrated. The residue was purified by silica gel, eluted with EA/PE (1:3~1:1) to afford *tert*-butyl (2-(2-(2-hydroxyethoxy)ethoxy)ethyl)carbamate (**7-4**, 45 g, 54 %) as a colorless oil.

$^1$HNMR ($CDCl_3$, 400 MHz) δ (ppm) 3.76-3.55 (m, 10H), 3.33 (d, J = 4.8 Hz, 2H), 1.45 (s, 9H)

*7.5 Synthesis of tert-butyl (2-(2-(2-(4-(2-phenyl-5,7-bis(trifluoromethyl)pyrazolo[1,5-a]pyrimidin-3-yl)phenoxy)ethoxy)ethoxy)ethyl)carbamate (**7-5**)*

To a solution of *tert*-butyl (2-(2-(2-hydroxyethoxy)ethoxy)ethyl)carbamate (**7-4**, 20 g, 81 mmol) in tetrahydrofuran (0.6 L) were added 4-[2-phenyl-5,7-*bis*(trifluoromethyl)pyrazolo[1,5-*a*]pyrimidin-3-yl]phenol (PHTPP, 34 g, 81 mmol), $PPh_3$ (32 g, 121 mmol) and diisopropyl azodicarboxylate (DIAD, 24.5 g, 121 mmol). The mixture was stirred at 65 $^oC$ for 16 hours. The solution was concentrated. The residue was purified by silica gel, eluted with dichloromethane/methanol (100:1~50:1) to afford crude *tert*-butyl (2-(2-(2-(4-(2-phenyl-5,7-bis(trifluoromethyl)pyrazolo[1,5-*a*]pyrimidin-3-yl)phenoxy)ethoxy)ethoxy)ethyl)carbamate (**7-5**, 20 g, 38 %) as a yellow solid.

$^1$H-NMR (DMSO_$d_6$, 400 MHz) δ (ppm) 7.70-7.42 (m, 9H), 7.12 (d, J = 8.8 Hz, 1H), 4.83 (t, J = 6.4 Hz, 1H), 4.20 (t, J = 4.4 Hz, 1H), 3.83 (t, J = 4.8 Hz, 1H), 3.65 (q, J = 2.4 Hz, 1H), 3.59 (t, J = 2.4 Hz, 1H), 3.45 (t, J = 6 Hz, 1H), 3.13 (q, J = 6 Hz, 1H), 1.42 (s, 6H), 1.24 (d, J = 6.4

Hz, 9H) ; ESI-MS Calcd mz for $C_{31}H_{32}F_6N_4O_5$ $[M+Na]^+$ 677.50 Found 677.23

*7.6 Synthesis of 2-(2-(2-(4-(2-phenyl-5,7-bis(trifluoromethyl)pyrazolo[1,5-a]pyrimidin-3-yl)phenoxy)ethoxy)ethoxy)ethan-1-amine (**7-6**)*

To a solution of tert-butyl (2-(2-(2-(4-(2-phenyl-5,7-bis(trifluoromethyl)pyrazolo[1,5-*a*]pyrimidin-3-yl)phenoxy)ethoxy)ethoxy)ethyl)carbamate (**7-5**, 20 g, 31 mmol) in Hydrochloric acid/Ethyl acetate (300 mL), the mixture was stirred at r.t for 2 hours. The reaction was concentrated to afford crude 2-(2-(2-(4-(2-phenyl-5,7-bis(trifluoromethyl)pyrazolo[1,5-*a*]pyrimidin-3-yl)phenoxy)ethoxy)ethoxy)ethan-1-amine (**7-6**, 15 g, 88 %) as a yellow solid.

ESI-MS Calcd mz for $C_{26}H_{24}F_6N_4O_3$ $[M+Na]^+$ 555.20 Found 555.18

*7.7 Synthesis of (R)-1-(4-(benzyloxy)-3-(3-phenylpropoxy)phenoxy)-3-((2-(2-(2-(4-(2-phenyl-5,7-bis(trifluoromethyl)pyrazolo[1,5-a]pyrimidin-3-yl)phenoxy)ethoxy)ethoxy)ethyl)amino)propan-2-ol (**PHTPP-1304**)*

To a solution of 2-(2-(2-(4-(2-phenyl-5,7-bis(trifluoromethyl)pyrazolo[1,5-*a*]pyrimidin-3-yl)phenoxy)ethoxy)ethoxy)ethan-1-amine (**7-6**, 27 g, 49 mmol) in methanol (500 mL) was added (R)-2-((4-(benzyloxy)-3-(3-phenylpropoxy)phenoxy)methyl)oxirane (**7-3**, 19 g, 49 mmol). The mixture was stirred at 65 $^oC$ for 16 hours. Then the reaction was concentrated. 4 batches were taken for this reaction. All the residue was gathered and purified by silica gel, eluted with dichloromethane/methanol (100:1~50:1) to afford crude (20 g), further purified by Pre-HPLC to afford (*R*)-1-(4-(benzyloxy)-3-(3-phenylpropoxy)phenoxy)-3-((2-(2-(2-(4-(2-phenyl-5,7-bis(trifluoromethyl)pyrazolo[1,5-*a*]pyrimidin-3-yl)phenoxy)ethoxy)ethoxy)ethyl)amino)propan-2-ol (**PHTPP-1304**, 10 g, 22 %) as a yellow solid.

$^1$H-NMR (DMSO_d$_6$, 500 MHz) δ (ppm) 8.10 (s, 1H), 7.67 – 7.60 (m, 2H), 7.46 (ddd, J = 13.6, 7.3, 5.5 Hz, 5H), 7.42 – 7.33 (m, 4H), 7.33 – 7.23 (m, 3H), 7.23 – 7.14 (m, 3H), 7.10 – 7.02 (m, 2H), 6.91 (d, J = 8.8 Hz, 1H), 6.57 (d, J = 2.8 Hz, 1H), 6.40 (dd, J = 8.8, 2.8 Hz, 1H), 5.01 (s, 2H), 4.19 – 4.11 (m, 2H), 3.96 (t, J = 6.3 Hz, 2H), 3.89 – 3.75 (m, 5H), 3.62 (dd, J = 5.9, 3.5 Hz, 2H), 3.56 (dd, J = 5.9, 3.5 Hz, 2H), 3.54 – 3.46 (m, 2H), 2.79 – 2.72 (m, 2H), 2.72 – 2.65 (m, 3H), 2.59 (dd, J = 11.8, 6.5 Hz, 1H), 2.01 (tt, J = 12.8, 6.3 Hz, 2H) ; $^{13}$C-NMR (DMSO_d$_6$, 125 MHz) δ (ppm)158.21, 154.80, 153.89, 149.85, 146.05, 145.01, 144.71, 142.09, 141.43, 137.73, 134.13, 133.82, 131.54, 131.07, 129.47, 128.84, 128.58, 128.32, 128.27, 127.63, 127.44, 125.81, 121.88, 121.39, 120.25, 119.20, 118.07, 116.36, 114.78, 111.47, 104.70, 104.30, 102.16, 71.26, 71.06, 70.22, 69.93, 69.72, 68.97, 68.23, 67.42, 67.16, 54.94, 52.52, 49.01, 31.48, 30.54 ; HRMS Calcd m/z for $C_{51}H_{50}F_6N_4O_7$ $[M+H]^+$ 945.3656 Found 945.3653

**Scheme 8.** Synthesis of (3*R*,4*S*,5*S*,6*R*)-5-methoxy-4-((2*R*,3*R*)-2-methyl-3-(3-methylbut-2-en-1-yl)oxiran-2-yl)-1-oxaspiro[2.5]octan-6-yl (13*E*,15*E*,17*E*,19*E*)-1-(3-(benzyloxy)phenyl)-12-oxo-5,8-dioxa-2,11-diazahenicosa-13,15,17,19-tetraen-21-oate (**Fumagillin-105**)

*8.1 Synthesis of tert-butyl (2-(2-(2-((3-(benzyloxy)benzyl)amino)ethoxy)ethoxy)ethyl)carbamate (**8**).*

To a solution of 3-(benzyloxy)benzaldehyde (**1-1**, 3 g, 14.13 mmol) in methanol (30 mL) was added 2,2'-(ethane-1,2-diylbis(oxy))diethanamine (5.26 g, 21.20 mmol). The mixture was stirred for 6 hours at 25 °C. $NaBH_3CN$ (1.78 g, 28.27 mmol) was added at r.t. The reaction mixture was stirred overnight at 50 $^0$C. The above solution was poured into water. The solution was extracted with ethyl acetate. The combined organic layers were washed with brine, dried over $Na_2SO_4$, filtered and concentrated. The crude was purified by column chromatography on

silica gel (petroleum ether/ethyl ether = 3:1) to give *tert*-butyl (2-(2-(2-((3-(benzyloxy)benzyl)amino)ethoxy)ethoxy)ethyl)carbamate (**8**, 3.5 g, 56%) as yellow oil.

$^1$H-NMR (DMSO_d$_6$, 400 MHz) δ (ppm) 7.46-7.44 (m, 2H), 7.39 (t, J = 7.2 Hz, 1H), 7.34 - 7.33 (m, 2H), 7.25 (t, J = 8 Hz, 1H), 7.05 (m, 1H), 6.95 – 6.91 (m, 2H), 6.75 (brs, 1H), 5.09 (s, 2H), 3.82 (s, 2H), 3.53 – 3.48 (m, 6H), 3.37 (t, J = 6 Hz, 2H), 3.07 (q, J = 6 Hz, 2H), 2.76 (t, J = 5.6 Hz, 2H), 1.36 (s, 9H)

*8.2 Synthesis of 2-(2-(2-aminoethoxy)ethoxy)-N-(3-(benzyloxy)benzyl)ethan-1-amine (**8-1**).*

A mixture of *tert*-butyl (2-(2-(2-((3-(benzyloxy)benzyl)amino)ethoxy)ethoxy)ethyl)carbamate (**8**, 1 g, 2.25 mmol) in Hydrochloric acid / Ethyl acetate (10 mL, 2M) was stirred for 4 hours at 25 $^o$C. The mixture was concentrated under vacuum and poured into saturated $NaHCO_3$. The solution was extracted with ethyl acetate. The combined organic layers were washed with brine, dried over $Na_2SO_4$, filtered and concentrated to afford Synthesis of 2-(2-(2-aminoethoxy)ethoxy)-*N*-(3-(benzyloxy)benzyl)ethan-1-amine (**8-1**, 600 mg, 77%) as yellow oil.

$^1$H-NMR (DMSO_d$_6$, 400 MHz) δ (ppm) 7.46 – 7.46 (m, 2H), 7.43 (t, J = 7.2 Hz, 1H), 7.34 - 7.32 (m, 2H), 7.20 (t, J = 7.6 Hz, 1H), 6.99 (s, 1H), 6.89 – 6.84 (m, 2H), 5.08 (s, 2H), 3.67 (s, 2H), 3.51 (s, 4H), 3.46 (t, J = 6 Hz, 2H), 3.36 (t, J = 6 Hz, 2H), 2.65 (t, J = 5.6 Hz, 2H), 2.61 (t, J = 5.6 Hz, 2H)

*8.3 Synthesis of (3R,4S,5S,6R)-5-methoxy-4-((2R,3R)-2-methyl-3-(3-methylbut-2-en-1-yl)oxiran-2-yl)-1-oxaspiro[2.5]octan-6-yl (13E,15E,17E,19E)-1-(3-(benzyloxy)phenyl)-12-oxo-5,8-dioxa-2,11-diazahenicosa-13,15,17,19-tetraen-21-oate (**Fumagillin-105**)*

To a solution of 2-(2-(2-aminoethoxy)ethoxy)-*N*-(3-(benzyloxy)benzyl)ethan-1-amine (**8-1**, 140 mg, 0.44 mmol) in dichloromethane (4 mL) was added Fumagillin (200 mg, 0.44 mmol),

hydroxybenzotriazole (HOBT, 68 mg, 0.50 mmol), 1-ethyl-3-(3-dimethylaminopropyl)carbodiimide (EDCI (96 mg, 0.50 mmol) and triethylamine (88 mg, 0.88 mmol). The mixture was stirred at 30 °C for overnight. Then the reaction was concentrated. The residue was purified by Prep-HPLC twice to afford (3*R*,4*S*,5*S*,6*R*)-5-methoxy-4-((2*R*,3*R*)-2-methyl-3-(3-methylbut-2-en-1-yl)oxiran-2-yl)-1-oxaspiro[2.5]octan-6-yl(13*E*,15*E*,17*E*,19*E*)-1-(3-(benzyloxy)phenyl)-12-oxo-5,8-dioxa-2,11-diazahenicosa-13,15,17,19-tetraen-21-oate (**Fumagillin-105**, 21 mg, 4%) as a yellow solid.

$^{1}$H-NMR (DMSO_d$_6$, 400 MHz) δ (ppm) 8.15 (m, 1H), 7.45 – 7.32 (m, 5H), 7.32 (t, J = 3.6 Hz, 1H), 7.20 – 7.19 (m, 1H), 6.99 (s, 1H), 6.87 (t, J = 3.6 Hz, 3H), 6.70 (m, 1H), 6.61 – 6.58 (m, 2H), 6.15 (d, J = 14.8 Hz, 1H), 6.05 (d, J = 15.2 Hz, 1H), 5.57 (d, J = 2.4 Hz, 1H), 5.19 (m, 1H), 5.19 (s, 2H), 3.68 (s, 2H), 3.60 (dd, J = 10.8, 2.4 Hz, 1H), 3.50 (s, 4H), 3.48 – 3.43 (m, 4H), 3.28 (m, 5H), 2.87 (d, J = 4.4 Hz, 1H), 2.64 – 2.57 (m, 5H), 2.20 – 2.18 (m, 2H), 2.00 (m, 1H), 1.87 – 1.80 (m, 3H), 1.71 (s, 3H), 1.61 (s, 3H), 1.09 (s, 3H) ; $^{13}$C-NMR (DMSO_d$_6$, 100 MHz) δ (ppm) 165.37, 164.90, 158.36, 144.23, 142.63, 140.30, 138.23, 137.33, 137.20, 134.69, 133.67, 132.31, 129.62, 129.09, 128.37, 127.73, 127.61, 127.08, 121.87, 120.30, 119.31, 114.24, 112.80, 78.83, 70.09, 69.57, 69.56, 69.08, 66.43, 59.61, 59.46, 58.06, 56.00, 52.78, 50.47, 50.48, 47.98, 47.57, 35.11, 29.11, 29.01, 28.89, 28.81, 28.71, 28.67, 28.57, 26.92, 26.56, 25.47, 25.35, 25.09, 22.06, 17.81, 13.94 ; HRMS Calcd m/z for $C_{46}H_{61}N_2O_9$ $[M+H]^+$ 785.4379 Found 785.4372.

**Scheme 9.** Synthesis of (*R*)-*N*-(15-(3,4-bis(benzyloxy)phenoxy)-14-hydroxy-3,6,9-trioxa-12-azapentadecyl)-4-phenylbutanamide (**PBA-1106**)

*9.1 Synthesis of N-(2-(2-(2-(2-aminoethoxy)ethoxy)ethoxy)ethyl)-4-phenylbutanamide* ***(9-1)***

To a solution of *tert*-butyl (2-(2-(2-(2-aminoethoxy)ethoxy)ethoxy)ethyl)carbamate (**9**, 2.92 g, 10 mmol) and 4-phenylbutanoic acid (**9-1**, 1.64 g, 10 mmol) in dimethylformamide (20 mL) was added Hexafluorophosphate Azabenzotriazole Tetramethyl Uronium (HATU, 5.7 g, 15 mmol) at 0 $^oC$. The solution was allowed to warm to r.t. for overnight. After TLC monitoring, the solution was diluted with water and extracted with ethyl acetate. The organic solution was dried over $Na_2SO_4$ and concentrated to dryness to give colorless oil, which was dissolved in dichloromethane (30 mL). The solution was added trifluoroacetic acid (10 mL) at 0 $^oC$ and then allowed to warm to r.t for 4 hours, then water was added. The bi-phase solution was separated with funnel. The aqueous phase was neutralized with $Na_2CO_3$ solution to pH = 9. And then extracted with dichloromethane. The organic layers were collected and concentrated, purified with flash column to give *N*-(2-(2-(2-(2-aminoethoxy)ethoxy)ethoxy)ethyl)-4-phenylbutanamide (**9-1**, 1.3 g, 38%).

ESI-MS Calcd m/z for $C_{18}H_{30}N_2O_4$ $[M+H]^+$ 339.22 Found 339.20

*9.2 Synthesis of 3,4-bis(benzyloxy)phenol (**9-2**)*

To a solution of 3,4-bis(benzyloxy)benzaldehyde (**5-1**, 120 g, 377 mmol) in dichloromethane (1000 mL) was added meta-chloroperoxybenzoic acid (mCPBA,129.6 g, 754 mmol). The mixture was stirred at r.t for 16 hours. Then the reaction was filtered. The filtration was concentrated. The residue was purified by silica gel, eluted with ethyl acetate/petroleum ether (1:15~1:5) to afford 3,4-bis(benzyloxy)phenol (**9-2**, 62 g, 54 %) as an off-white solid.

$^1H$-NMR (DMSO_$d_6$, 400 MHz) δ (ppm) 9.03 (s, 1H), 7.46 – 7.32 (m, 10H), 6.83 (d, J = 8.4 Hz, 1H), 6.49 (s, 1H), 6.25 (m, 1H), 5.07 (s, 2H), 4.98 (s, 2H)

*9.3 Synthesis of (R)-2-((3,4-bis(benzyloxy)phenoxy)methyl)oxirane (**9-3**)*

To a solution of 3,4-bis(benzyloxy)phenol (**9-2**, 3.06 g, 10 mmol) and (*R*)-2-

(chloromethyl)oxirane (0.92 g, 10 mmol) in dimethylformamide (50 mL) was added $Cs_2CO_3$ (6.5 g, 20 mmol) at r.t. The mixture was stirred at r.t overnight, diluted with water. The aqueous phase was extracted with ethyl acetate. The combined organic layer was washed with water and brine, dried and concentrated. The crude material was purified by silica gel column (petroleum ether/ethyl acetate = 20/1) to give (*R*)-2-((3,4-bis(benzyloxy)phenoxy)methyl)oxirane (**9-3**, 0.84 g, 23%).

ESI-MS Calcd m/z for $C_{23}H_{22}O_4$ $[M+Na]^+$ 385.15 Found 385.20

*9.4 Synthesis of (R)-N-(15-(3,4-bis(benzyloxy)phenoxy)-14-hydroxy-3,6,9-trioxa-12-azapentadecyl)-4-phenylbutanamide (**PBA-1106**)*

A solution of *N*-(2-(2-(2-(2-aminoethoxy)ethoxy)ethoxy)ethyl)-4-phenylbutanamide (**9-1**, 670 mg, 2 mmol) and (*R*)-2-((3,4-bis(benzyloxy)phenoxy)methyl)oxirane (**9-3**, 720 mg, 2 mmol) in ethanol (30 mL) was heated to reflux for 6 hours. The solution was concentrated and purified prep-HPLC to give (*R*)-*N*-(15-(3,4-bis(benzyloxy)phenoxy)-14-hydroxy-3,6,9-trioxa-12-azapentadecyl)-4-phenylbutanamide (**PBA-1106**) as pale yellow oil (56 mg, 4%).

$^1$H-NMR (DMSO_d$_6$, 400 MHz) δ (ppm) 7.83 (t, J = 5.2 Hz, 1H), 7.46 – 7.25 (m, 12H), 7.15 (dd, J = 14.4, 7.2 Hz, 3H), 6.92 (d, J = 8.8 Hz, 1H), 6.67 (d, J = 5.6 Hz, 1H), 6.41 (dd, J = 8.4, 2.4 Hz, 1H), 5.11 (s, 2H), 5.02 (s, 2H), 4.95 (brs, 1H), 3.86 – 3.78 (m, 3H), 3.49 – 3.35 (m, 13H), 3.18 (q, J = 5.6 Hz, 2H), 2.69 – 2.57 (m, 6H), 2.07 (t, J = 7.2 Hz, 2H), 1.81 – 1.73 (m, 2H) ; $^{13}$C-NMR (DMSO_d$_6$, 100 MHz) δ (ppm) 171.79, 153.66, 149.35, 142.18, 141.69, 137.54, 137.14, 128.28, 128.19, 128.18, 128.15, 127.65, 127.55, 127.47, 127.37, 125.61, 116.27, 105.08, 102.78, 71.17, 71.03, 70.00, 69.92, 69.68, 69.66, 69.56, 69.47, 69.06, 68.07, 52.36, 48.86, 38.85, 38.38, 34.69, 36.55, 26.97 ; HRMS Calcd m/z for $C_{41}H_{52}N_2O_8$ $[M+H]^+$ 701.3815 Found 701.3802

**Scheme 10**. *N*-(3,5-dichlorophenyl)-*N*-((2-(2-(2-(((*R*)-3-(3,4-diphenethoxyphenoxy)-2-hydroxypropyl)amino)ethoxy)ethoxy)ethyl)carbamoyl)-2-hydroxy-2-methylbut-3-enamide (**VinclozolinM2-2204**)

*10.1 Synthesis of ethyl 2-hydroxy-2-methylbut-3-enoate (**10-1**)*

To a solution of ethyl 2-oxopropanoate (**10**, 5.00 g, 43.1 mmol) in tetrahydrofuran (25 mL) was added vinylmagnesium chloride (25.8 mL, 51.5 mmol, 2M in tetrahydrofuran) at -75 $^{0}$C under $N_2$ atmosphere. The mixture was stirred for 2 hours at -75 $^{0}$C. The mixture was poured into ice-water and extracted with ethyl acetate. The combined organic layers were washed with brine, dried over $Na_2SO_4$ and concentrated. The residue was purified by column chromatography (petroleum ether : ethyl acetate=100:1) to afford ethyl 2-hydroxy-2-methylbut-3-enoate (**10-1**, 2.5 g, 40.3 %) as yellow oil.

$^{1}$H-NMR ($CDCl_3$, 400 MHz) δ (ppm) 6.02 (dd, J = 17.6, 10.8 Hz, 1H), 5.48 (dd, J = 16.8, 0.8 Hz, 1H), 5.19 (dd, J = 10.4, 0.8 Hz, 1H), 4.29 – 4.24 (m, 2H), 1.48 (s, 3H), 1.35 – 1.24 (m, 4H)

*10.2 Synthesis of 3-(3,5-dichlorophenyl)-5-methyl-5-vinyloxazolidine-2,4-dione (**10-2**)*

A mixture of ethyl 2-hydroxy-2-methylbut-3-enoate (**10-1**, 2.50 g, 17.4 mmol), 1, 3-dichloro-5-isocyanatobenzene (4.88 g, 26.1 mmol) and triethylamine (5.27 g, 52.2 mmol) in toluene (50 mL) was stirred for 4 hours at 100 $^{o}$C. Then the reaction was concentrated. The residue was triturated with tert-butyl methyl ether (MTBE, 20 mL) and filtered. The filter cake was dried to afford Synthesis of 3-(3,5-dichlorophenyl)-5-methyl-5-vinyloxazolidine-2,4-dione (**10-2**, 1.00 g, 20.1 %) as a white solid.

$^{1}$H-NMR ($CDCl_3$, 400 MHz) δ (ppm) 7.47 – 7.44 (m, 3H) , 6.04 (dd, J = 17.2, 10.8 Hz, 1H),

5.64 (d, J = 17.2 Hz, 1H), 5.45 (d, J = 10.8 Hz, 1H), 1.79 (s, 3H) ; ESI-MS Calcd m/z for $C_{12}H_9Cl_2NO_3$ $[M+H]^+$ 285.99 Found 286.00

*10.3 Synthesis of (R)-1-((2-(2-(2-aminoethoxy)ethoxy)ethyl)amino)-3-(3,4-diphenethoxyphenoxy)propan-2-ol* ***(10-3)***

A mixture of (*R*)-2-((3,4-diphenethoxyphenoxy)methyl)oxirane (**2-3**, 1.1 g, 2.82 mmol) and 2,2'-(ethane-1,2-diylbis(oxy))diethanamine (0.835 g, 5.64 mmol) in acetonitrile (22 mL) was stirred overnight at 70 $^oC$. Then the reaction was concentrated. The residue was purified by prep-HPLC to afford (*R*)-1-((2-(2-(2-aminoethoxy)ethoxy)ethyl)amino)-3-(3,4-diphenethoxyphenoxy)propan-2-ol (**10-3**, 0.50 g, 32.9 %) as yellow oil.

$^1H$-NMR ($CDCl_3$, 400 MHz) δ (ppm) 8.46 (s, 2H), 7.34 - 7.28 (m, 8H), 7.26 – 7.21 (m, 2H), 6.76 (d, J = 8.4 Hz, 1H), 6.50 (d, J = 2.8 Hz, 1H), 6.35 (dd, J = 9.2, 2.4 Hz, 1H), 4.4 (brs, 1H), 4.165 – 4.09 (m, 4H), 3.97 (d, J = 7.6 Hz, 1H), 3.89 – 3.88 (m, 1H), 3.78 (brs, 2H), 3.67 – 3.50 (m, 7H), 3.23 – 3.07 (m, 10H) ; ESI-MS Calcd m/z for $C_{31}H_{42}N_2O_6$ $[M+H]^+$ 538.30 Found 538.90

*10.4 Synthesis of N-(3,5-dichlorophenyl)-N-((2-(2-(2-(((R)-3-(3,4-diphenethoxyphenoxy)-2-hydroxypropyl)amino)ethoxy)ethoxy)ethyl)carbamoyl)-2-hydroxy-2-methylbut-3-enamide* ***(VinclozolinM2-2204)***

To a solution of 3-(3,5-dichlorophenyl)-5-methyl-5-vinyloxazolidine-2,4-dione (**10-2,** 177 mg, 0.619 mmol) and (*R*)-1-((2-(2-(2-aminoethoxy)ethoxy)ethyl)amino)-3-(3,4-diphenethoxyphenoxy)propan-2-ol (**10-3**, 500 mg, 0.929 mmol) in dimethylchloride (10 mL) was added triethylamine (313 mg, 3.10 mmol) at r.t. The mixture was stirred at 40 $^oC$ for 16 hours. Then the reaction was concentrated. The residue was purified by prep-HPLC to afford *N*-(3,5-dichlorophenyl)-*N*-((2-(2-(2-(((*R*)-3-(3,4-diphenethoxyphenoxy)-2-

hydroxypropyl)amino)ethoxy)ethoxy)ethyl)carbamoyl)-2-hydroxy-2-methylbut-3-enamide (**VinclozolinM2-2204**, 30 mg) as a yellow solid.

$^1$H-NMR ($CD_3OD$, 400 MHz) δ (ppm) 7.45 (s, 2H), 7.35-7.28 (m, 8H), 7.25-7.14 (m, 2H), 7.07 (s, 1H), 6.83 (d, J = 8.8 Hz, 1H), 6.59 (d, J = 2.4 Hz, 1H), 6.44 (dd, J = 8.8, 2.8 Hz, 1H), 6.28-6.23 (m, 1H), 5.46 (d, J = 6 Hz, 1H), 5.30 (d, J = 10.8 Hz, 1H), 4.17 (t, J = 10 Hz, 3H), 4.08 (t, J = 6.8 Hz, 2H), 3.98-3.93 (m, 2H), 3.72 (t, J = 4.8 Hz, 2H), 3.62 (s, 4H), 3.56-3.51 (m, 3H), 3.46-3.43 (m, 2H), 3.33-3.25 (m, 3H), 3.19 (t, J = 9.6 Hz, 3H), 3.17 (t, J = 6 Hz, 2H), 1.70 (s, 3H) ; $^{13}$C-NMR (DMSO_$d_6$, 100 MHz) δ (ppm) 170.71, 153.86, 151.77, 149.94, 142.88, 142.33, 142.10, 139.09, 138.96, 138.52, 134.65, 134.32, 129.48, 129.45, 128.68, 126.68, 126.62, 122.09, 118.66, 116.66, 116.23, 116.11, 105.65, 102.71, 81.79, 71.12, 70.66, 70.07, 69.96, 69.89, 69.48, 69.14, 68.08, 66.82, 51.43, 48.09, 35.83, 35.59, 23.09, 22.82, 22.77 ; HRMS Calcd m/z for $C_{43}H_{51}Cl_2N_3O_9$ $[M+H]^+$ 824.3075 Found 824.3075

**Scheme 11**. *N*-(1-(3,4-bis(benzyloxy)phenyl)-5,8,11,14,17,20,23-heptaoxa-2-azapentacosan-25-yl)-4-phenylbutanamide (**PBA-1105b**)

*11.1 Synthesis of 23-hydroxy-3,6,9,12,15,18,21-heptaoxatricosyl 4-methylbenzenesulfonate **(11-1)***

To a mixture of 3,6,9,12,15,18,21-heptaoxatricosane-1,23-diol (11, 5.00 g, 13.5 mmol) in dichloromethane (30 mL) was added triethylamine (1.36 g, 13.5 mmol) followed by tosyl chloride (1.28 g, 6.75 mmol) at 0 °C. The mixture was stirred at 0 °C for 30 min, washed with water. The combined organic layers were dried over sodium sulfate, filtered and concentrated. The crude was purified by column chromatography (dichloromethane/methanol=50/1~5/1) to give 23-hydroxy-3,6,9,12,15,18,21-heptaoxatricosyl 4-methylbenzenesulfonate (**11-1**, 1.80 g,

50.7 %) as yellow oil.

$^{1}$H-NMR ($CDCl_3$, 400 MHz) δ (ppm) 7.81 (d, J = 8 Hz, 2H), 7.36 (d, J = 8.4 Hz, 2H), 4.16 (t, J = 4.4 Hz, 2H), 3.74-3.59 (m, 32H), 2.61 (brs, 1H), 2.09 (s, 3H) ; ESI-MS Calcd m/z for C23H40O11S [M+H]+ 525.23 Found 252.20

*11.2 Synthesis of 23-amino-3,6,9,12,15,18,21-heptaoxatricosan-1-ol (**11-2**)*

To a solution of 23-hydroxy-3,6,9,12,15,18,21-heptaoxatricosyl 4-methylbenzenesulfonate (**11-1**, 1.80 g, 3.43 mmol) and ammonium chloride (0.92 g, 17.1 mol) in ammonium hydroxide (20 mL) then stirred at 40 °C overnight. The mixture was cooled to r.t and concentrated. Then stirred with dichloromethane (30 mL*3), filtered and concentrated to give 23-amino-3,6,9,12,15,18,21-heptaoxatricosan-1-ol (**11-2**, 1.30 g, crude) as a yellow oil.

ESI-MS Calcd m/z for $C_{16}H_{35}NO_8$ $[M+H]^+$ 370.24 Found 370.20

*11.3 Synthesis of tert-butyl (23-hydroxy-3,6,9,12,15,18,21-heptaoxatricosyl)carbamate (**11-3**)*

To a stirred solution of 23-amino-3,6,9,12,15,18,21-heptaoxatricosan-1-ol (**11-2**, 1.30 g, crude, 3.52 mmol) and sodium hydroxide (0.169 g, 4.22 mmol) in 1,4 dioxane (15 mL) and water (15 mL) was added di-tert-butyl pyrocarbonate (0.920 g, 4.22 mmol) dropwise at 10 oC. The resulting reaction mixture was allowed to r.t and stirred for 16 hours. The reaction mixture was transferred into ice water and the resulting mixture was extracted using ethyl acetate. The combined organic layers were dried over anhydrous sodium sulfate and filtered. The filtrate was evaporated under reduced pressure to give *tert*-butyl (23-hydroxy-3,6,9,12,15,18,21-heptaoxatricosyl)carbamate (**11-3**, 1.00 g, crude)

$^{1}$H-NMR ($CDCl_3$, 400 MHz) δ (ppm) 3.74 (t, J = 4 Hz, 2H), 3.69-3.62 (m, 26H), 3.55 (t, J = 5.2 Hz, 2H), 1.46 (s, 9H)

*11.4 Synthesis of 2,2-dimethyl-4-oxo-3,8,11,14,17,20,23,26-octaoxa-5-azaoctacosan-28-yl 4-methylbenzenesulfonate (**11-4**)*

To a solution of *tert*-butyl (23-hydroxy-3,6,9,12,15,18,21-heptaoxatricosyl)carbamate (**11-3**, 1.00 g, crude, 2.13 mmol) in dichloromethane (10 mL) was added triethylamine (0.323 g, 3.19 mmol) followed by tosyl chloride (0.607 g, 3.19 mmol) at 0 $^{o}$C. The mixture was stirred at 25 $^{o}$C for overnight, washed with water. The combined organic layers were dried over sodium sulfate, filtered and concentrated. The crude was purified by column chromatography (dichloromethane/methanol=50/1~5/1) to give 2,2-dimethyl-4-oxo-3,8,11,14,17,20,23,26-octaoxa-5-azaoctacosan-28-yl 4-methylbenzenesulfonate (**11-4**, 1.00 g, crude) as yellow oil.

$^{1}$H-NMR ($CDCl_3$, 400 MHz) δ (ppm) 7.81 (d, J = 8.4 Hz, 2H), 7.35 (d, J = 8 Hz, 2H), 5.32 (brs, 1H), 4.17 (t, J = 4.8 Hz, 2H), 3.71-3.53 (m, 29H), 3.32 (m, 2H), 2.46 (s, 3H), 1.46 (s, 9H)

*11.5 Synthesis of tert-butyl (23-azido-3,6,9,12,15,18,21-heptaoxatricosyl)carbamate (**11-5**)*

To a solution of 2,2-dimethyl-4-oxo-3,8,11,14,17,20,23,26-octaoxa-5-azaoctacosan-28-yl 4-methylbenzenesulfonate (**11-4**, 1.00 g, crude, 1.60 mmol) in *N,N*-Dimethylformamide (30 mL) was added sodium azide ($NaN_3$, 0.208 g, 3.20 mmol). The reaction mixture was then stirred at 85 $^{o}$C for overnight. One completion, the solvent was quenched with water, extracted with ethyl acetate and combine the organic phase. The solution was used in the next step directly.

$^{1}$H-NMR ($CDCl_3$, 400 MHz) δ (ppm) 8.36 (d, J = 8.8 Hz, 2H), 8.10 (d, J = 8.4 Hz, 2H), 7.84 (d, J = 8.8 Hz, 2H), 7.37-7.43 (m, 5H), 7.03 (d, J = 8.4 Hz, 2H), 5.86 (t, J = 4.0 Hz, 1H), 5.16 (s, 2H), 4.49 (d, J = 4.0 Hz, 2H)

*11.6 Synthesis of tert-butyl (23-amino-3,6,9,12,15,18,21-heptaoxatricosyl)carbamate (**11-6**)*

To a stirred suspension of *tert*-butyl (23-azido-3,6,9,12,15,18,21-heptaoxatricosyl)carbamate (**11-5,** ethyl acetate solution) was added 10% Pd/C (0.3 g) and stirred at 25 ºC for overnight under a hydrogen pressure of 50 psi. Then the Pd/C was filtered and concentrated at reduced pressure to give *tert*-butyl (23-amino-3,6,9,12,15,18,21-heptaoxatricosyl)carbamate (**11-6**, 0.400 g, 53.2 %) as yellow solid.

$^{1}$H-NMR ($CDCl_3$, 400 MHz) δ (ppm) 4.74 (brs, 1H), 3.67-3.62 (m, 24H), 3.57-3.51 (m, 4H), 3.33-3.32 (m, 2H), 2.90-2.87 (m, 2H), 1.47 (s, 9H) ; ESI-MS Calcd m/z for $C_{21}H_{44}N_2O_9$ $[M+H]^+$ 469.30 Found 469.30

*11.7 Synthesis of tert-butyl (25-oxo-28-phenyl-3,6,9,12,15,18,21-heptaoxa-24-azaoctacosyl)carbamate (**11-7**)*

To a solution of *tert*-butyl (23-amino-3,6,9,12,15,18,21-heptaoxatricosyl)carbamate (**11-6**, 0.400 g, 0.855 mmol) in dichloromethane (10 mL) was added triethylamine (27.6 g, 1.71 mmol), 4-phenylbutanoic acid (26.7 g, 1.03 mmol) and HATU (78.0 g, 1.28 mmol). The mixture was stirred at 25 ºC for overnight, washed with saturated sodium bicarbonate solution and sodium chloride saturated solution. The combined organic layers were dried over sodium sulfate, filtered and concentrated. The crude was purified by column chromatography (dichloromethane/methanol=50/1~5/1) to give *tert*-butyl (25-oxo-28-phenyl-3,6,9,12,15,18,21-heptaoxa-24-azaoctacosyl)carbamate (**11-7**, 0.450 g, 85.7 %) as yellow oil.

$^{1}$H-NMR ($CDCl_3$, 400 MHz) δ (ppm) 7.31-7.27 (m, 2H), 7.21-7.18 (m, 3H), 6.25 (brs, 1H), 5.06 (brs, 1H), 3.68-3.53 (m, 28H), 3.48-3.44 (m, 2H), 3.33-3.32 (m, 2H), 2.66 (t, J = 7.6 Hz, 2H) , 2.23 (t, J = 7.2 Hz, 2H) , 2.01-1.93 (m, 2H) , 1.45 (s, 9H) ; ESI-MS Calcd m/z for $C_{31}H_{54}N_2O_{10}$ $[M+H]^+$ 615.38 Found 615.40

*11.8 Synthesis of N-(23-amino-3,6,9,12,15,18,21-heptaoxatricosyl)-4-phenylbutanamide (**11-8**)*

To a solution of *tert*-butyl (25-oxo-28-phenyl-3,6,9,12,15,18,21-heptaoxa-24-azaoctacosyl)carbamate (**11-7**, 0.450 g, 0.732 mmol) and in ethyl acetate (10 mL) was add EA/HCl (4 N, 10 mL) slowly at 0 °C. Another batch was carried out as the above procedure. The mixture was stirred at 20 °C for 3 hours. The reaction was concentrated to give *N*-(23-amino-3,6,9,12,15,18,21-heptaoxatricosyl)-4-phenylbutanamide (**11-8**, 0.300 g, 79.5 %).

$^1$H-NMR ($CDCl_3$, 400 MHz) δ (ppm) 7.31-7.27 (m, 3H), 7.21-7.18 (m, 2H), 3.67-3.44 (m, 30H), 2.96 (t, J = 4.8 Hz, 2H), 2.67 (t, J = 7.6 Hz, 2H), 2.24 (t, J = 7.6 Hz, 3H) , 2.03-1.99 (m, 2H) ; ESI-MS Calcd m/z for $C_{26}H_{46}N_2O_8$ $[M+H]^+$ 515.33 Found 515.40

*11.9 Synthesis of N-(1-(3,4-bis(benzyloxy)phenyl)-5,8,11,14,17,20,23-heptaoxa-2-azapentacosan-25-yl)-4-phenylbutanamide (**PBA-1105b**)*

To a solution of *N*-(23-amino-3,6,9,12,15,18,21-heptaoxatricosyl)-4-phenylbutanamide (**11-8**, 0.050 g, 0.097 mmol) and 3,4-bis(benzyloxy)benzaldehyde (**5-1,** 0.039 g, 0.097 mmol) in methanol (10 mL) was stirred at 25 °C for 2 hours. Then sodium triacetoxyborohydride (0.062 g, 0.291 mmol) was added and stirred at 25 °C for 1 hour. Another 0.250 g of N-(23-amino-3,6,9,12,15,18,21-heptaoxatricosyl)-4-phenylbutanamide (**11-8**) were carried out as the above procedure. The reactions were concentrated together and purified by prep-TLC (dichloromethane/methanol=15/1) to give *N*-(1-(3,4-bis(benzyloxy)phenyl)-5,8,11,14,17,20,23-heptaoxa-2-azapentacosan-25-yl)-4-phenylbutanamide (**PBA-1105b**, 0.040 g, 8.40 %).

$^1$H-NMR ($CDCl_3$, 400 MHz) δ (ppm) 7.49-7.46 (m, 4H), 7.39-7.27 (m, 8H), 7.21-7.19 (m, 3H),

7.05 (s, 1H), 6.91-6.85 (m, 2H), 6.14 (brs, 1H), 5.17 (d, J = 12.8 Hz, 4H), 3.77 (s, 2H), 3.61 (s, 26H), 3.56 (t, J = 5.2 Hz, 2H), 3.47-3.43 (m, 2H), 2.79 (t, J = 5.2 Hz, 2H), 2.67 (t, J = 7.2 Hz, 2H), 2.20 (t, J = 7.2 Hz, 2H), 2.02-1.97 (m, 2H) ; $^{13}$C-NMR ($CDCl_3$, 100 MHz) δ (ppm) 172.77, 149.00, 147.99, 141.65, 137.45, 137.39, 133.51, 128.50, 128.44, 128.36, 127.74, 127.40, 127.31, 125.90, 121.24, 115.21, 115.19, 71.44, 71.23, 70.55, 70.51, 70.39, 70.30, 70.20, 69.87, 53.37, 48.46, 39.17, 35.76, 35.25, 27.16 ; HRMS Calcd m/z for $C_{47}H_{64}N_2O_{10}$ $[M+H]^+$ 817.4634 Found 817.4637

## SUPPLEMENTARY FIGURES

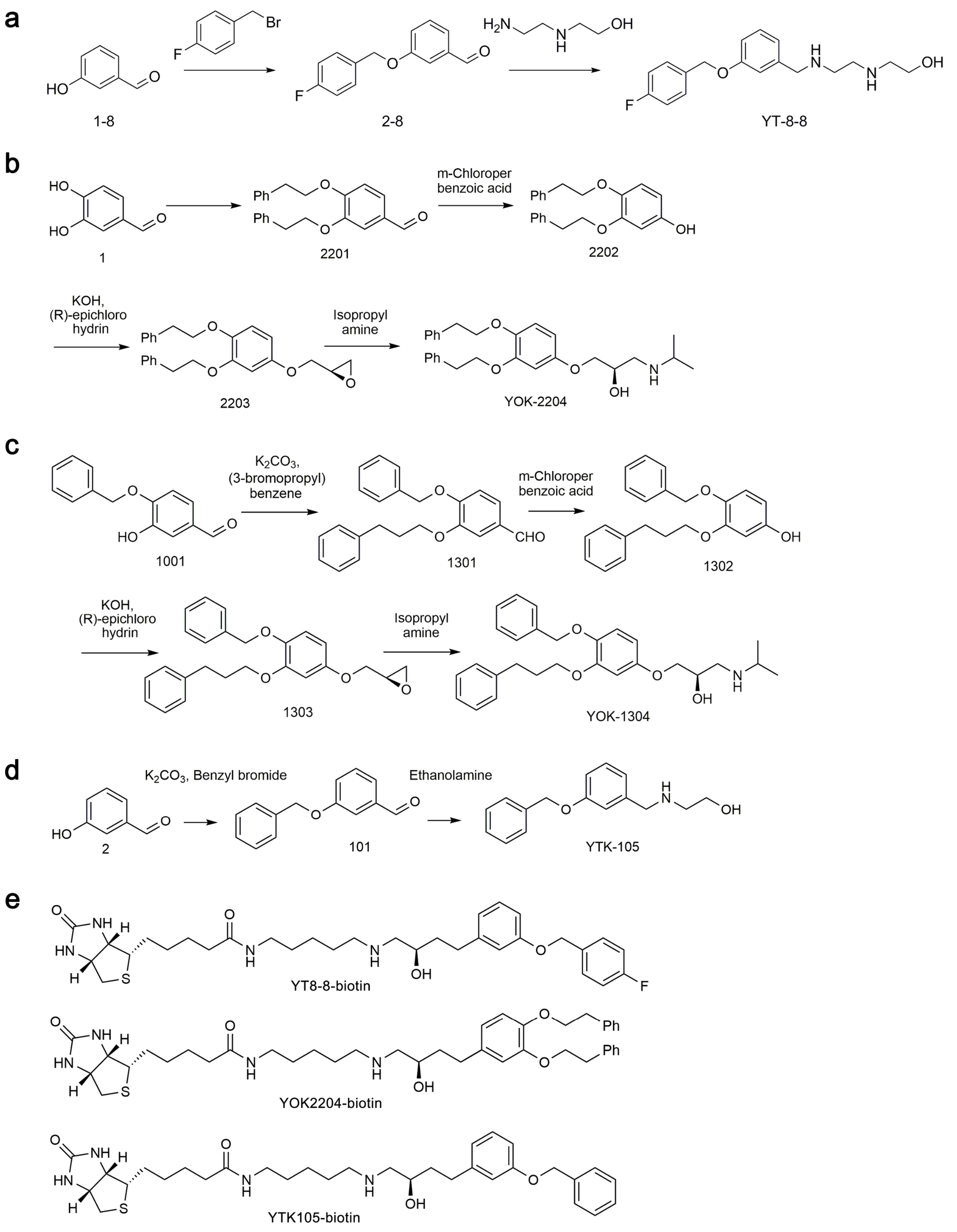


**Supplementary Fig. 1. Synthesis of small molecule ligands to p62 ZZ domain. (a,b,c,d,e)**

Synthesis of YT-8-8, YOK-2204, YOK-1304, YTK-105, and their biotinylated versions, respectively. Reagents and conditions are described in the Supplementary Methods.

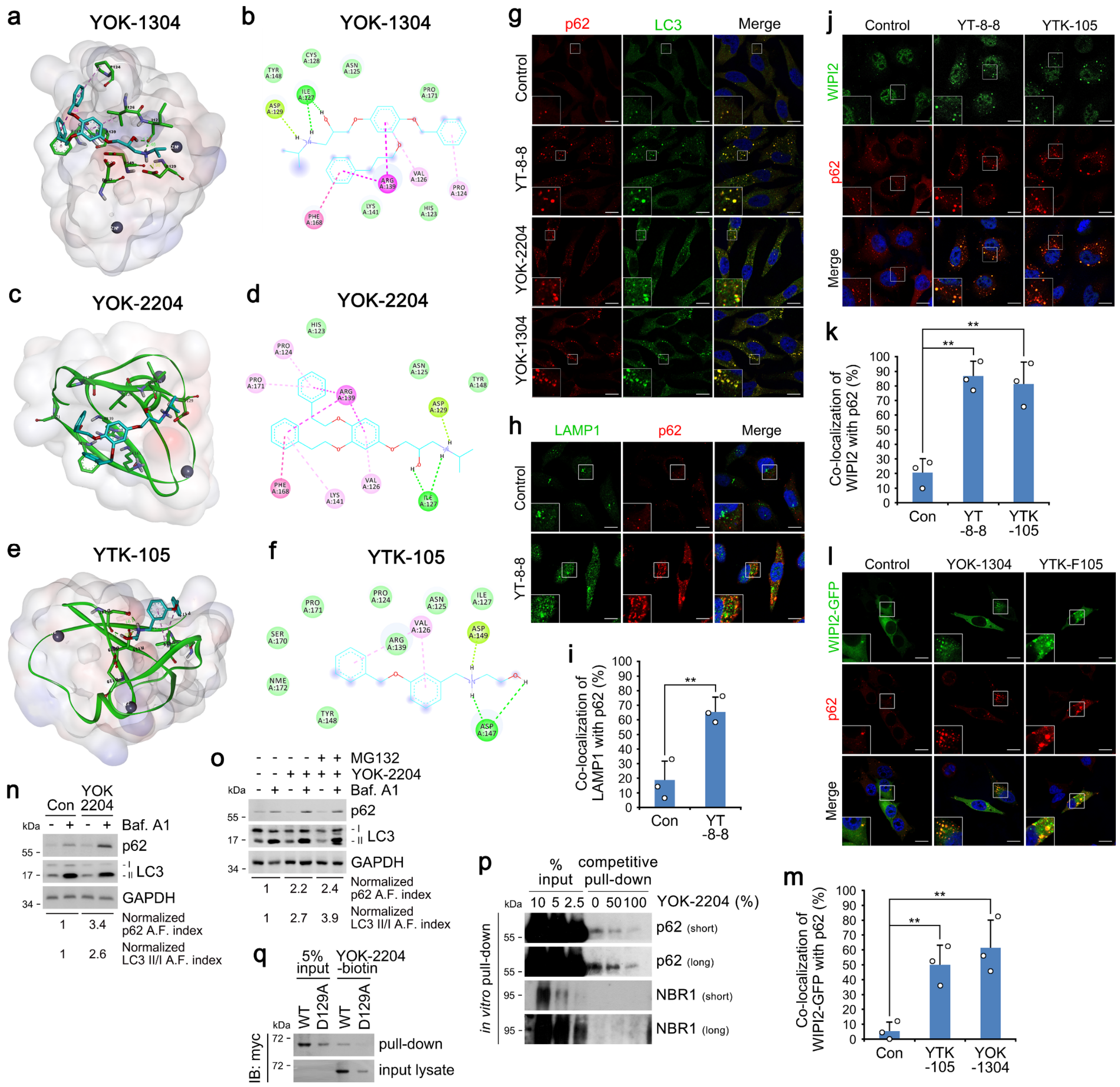

**Supplementary Fig. 2. Characterization of p62-ZZ ligands (a, c, e)** 3-D modeling of p62 ZZ ligands YOK-1304, YOK-2204 and YTK-105, respectively. **(b, d, f)** 2-D diagram for YOK-1304, YOK-2204 and YTK-105, respectively. **(g,h)** ICC of HeLa cells treated with the indicated compounds (2.5 µM, 24 h). Scale bar, 10 µm. **(i)** Quantification of **h** for co-localization (n=3, 50 cells). **(j)** ICC of HeLa cells treated with the indicated compounds (2.5 µM, 24 h). Scale bar, 10 µm. **(k)** Quantification of **j** for co-localization (n=3, 50 cells). **(l)** ICC of HeLa cells transiently expressing WIPI2-GFP and treated with the indicated compounds (2.5 µM, 6 h). **(m)** Quantification of **l** for co-localization (n=3, 50 cells). Scale bar, 10 µm. **(n)**

Autophagic flux assay in HeLa cells treated with YOK-2204 (2.5 µM, 24 h) in the presence or absence of HCQ (10 µM, 24 h). **(o)** Identical to **n**, but with YOK-2204 (2.5 µM, 24 h) in the presence or absence of HCQ (10 µM, 24 h) and/or MG132 (10 µM, 24 h). **(p)** Competition pulldown assay in HEK293T cells using biotinylated YOK-2204 in increasing presence of non-biotinylated YOK-2204. **(q)** Pulldown assay using biotinylated YOK-2204 in HEK293T cells transiently expressing wild-type or D129A p62-myc constructs. When indicated, n=3 biologically independent experiments each counting 50 cells. Data are presented as mean values ± SD where relevant. *P*-values (from a two-sided unpaired *t*-test): $^{**}P < 0.00825$ (for LAMP1-p62 colocalization), $^{**}P < 0.00407$ (for endogenous WIPI2-p62 colocalization), $^{**}P < 0.00590$ (for recombinant WIPI2-p62 colocalization). Source data are provided with this paper.

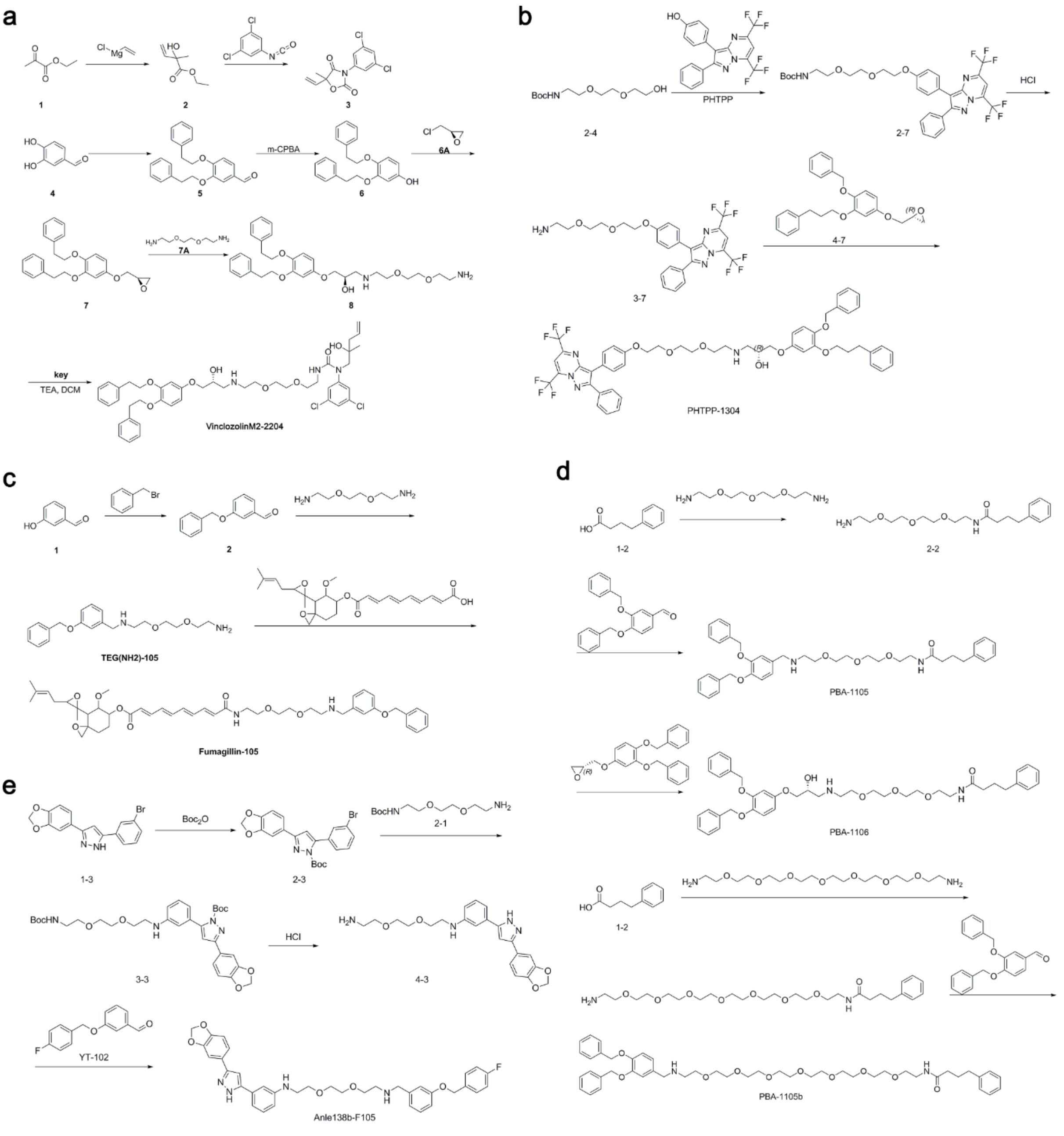


**Supplementary Fig. 3. Synthesis of chimeric protein degrader AUTOTAC (autophagy-targeting chimera) (a,b,c,d,e)** Synthesis of VinclozolinM2-2204, PHTPP-1304, Fumagilin-105, PBA-1105, -1106, -1105b and Anle138b-F105, respectively. Reagents and conditions are described in the Supplementary Methods.

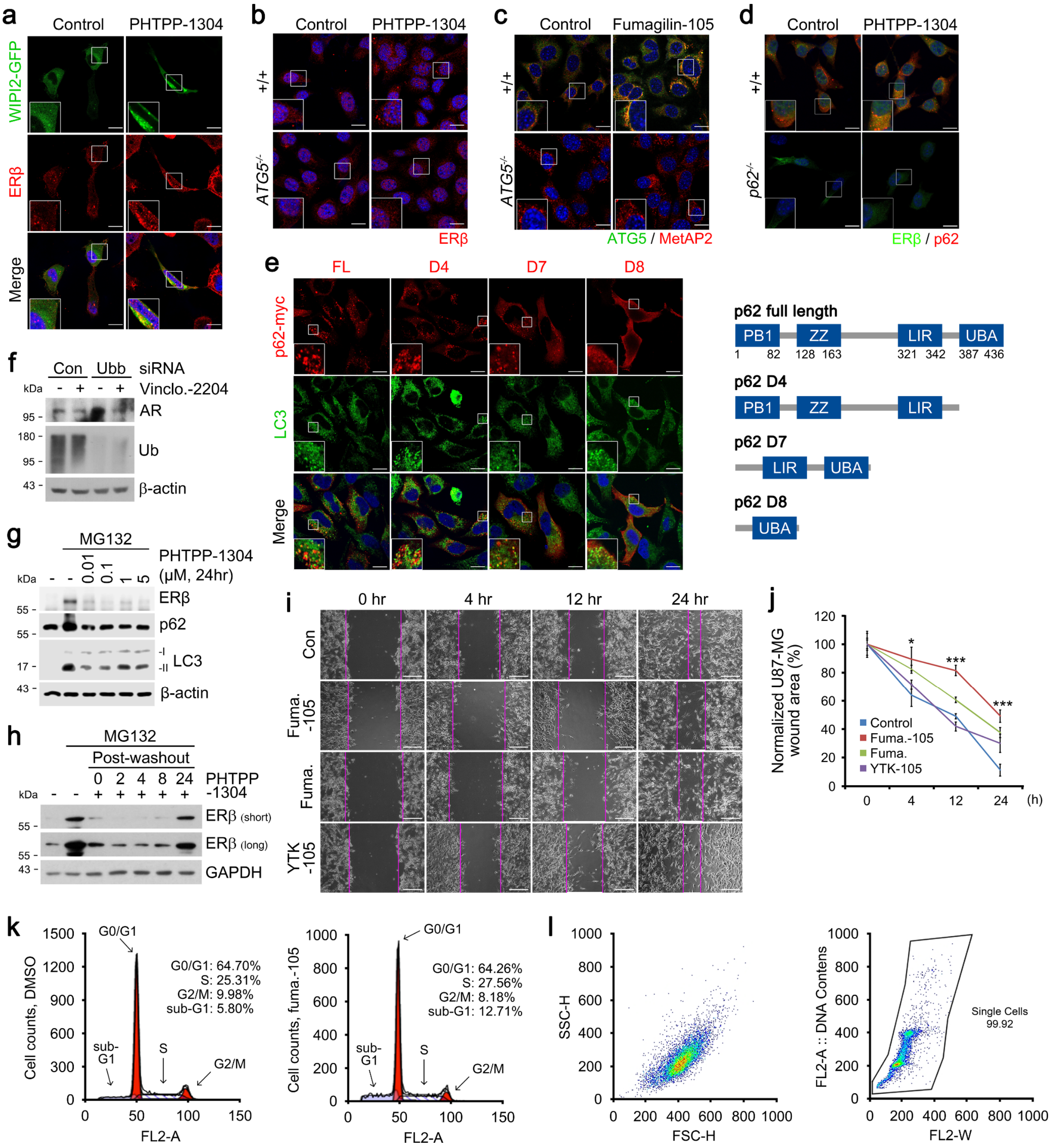


**Supplementary Fig. 4. Ub-independent oncoprotein-targeting AUTOTACs inhibit cancer cell growth and progression. (a)** ICC in HeLa cells transiently expressing WIPI2-GFP and treated with PHTPP-1304 (1 µM, 24 h). Scale bar, 10 µm. **(b, c)** ICC in wild-type or *ATG5*$^{-/-}$ MEF cells treated with PHTPP-1304 or Fumagillin-105, respectively (1 µM, 24 h). Scale bar, 10 µm. **(d)** ICC in wild type or *p62*$^{-/-}$ MEF cells treated with PHTPP-1304 (1 µM, 24 h). Scale bar, 10 µm. **(e)** ICC in HeLa cells transiently expressing full-length (FL) or domain-deleted

(D4, D7 or D8) p62-myc constructs as shown in schematic. Scale bar, 10 μm. **(f)** WB in HeLa cells treated with vinclozolinM2-2204 (0.1 μM, 24 h) under siRNA-mediated knockdown of *Ubb* (40 nM, 48 h). **(g)** WB in MCF7 cells treated with PHTPP-1304 with the indicated concentrations (24 h) and MG132 (1 μM, 24 h). **(h)** WB in MCF7 cells treated with MG132 (1 μM, 24 h) and PHTPP-1304 (500 nM, 24 h) with the culture medium subsequently washed out for the indicated durations. **(i)** Wound healing assay in U87-MG cells treated with Fumagillin-105, Fumagillin or YTK-105 (5 μM) at the indicated time points. Scale bar, 100 μm. **(j)** Quantification of **i** (n=3 biologically independent wounds measured). **(k)** Flow cytometry of HeLa cells treated with DMSO negative control or Fumagillin-105 (1 μM, 48 h). **(l)** Gating strategy for **k**. Data are presented as mean values ± SD where relevant. *P*-values (from a two-sided unpaired *t*-test): *$P < 0.0201$ (for Fuma.-105 at 4 hours), ***$P < 0.000177$ (for Fuma.-105 at 12 hours), ***$P < 0.000421$ (for Fuma.-105 at 24 hours). Source data are provided with this paper.

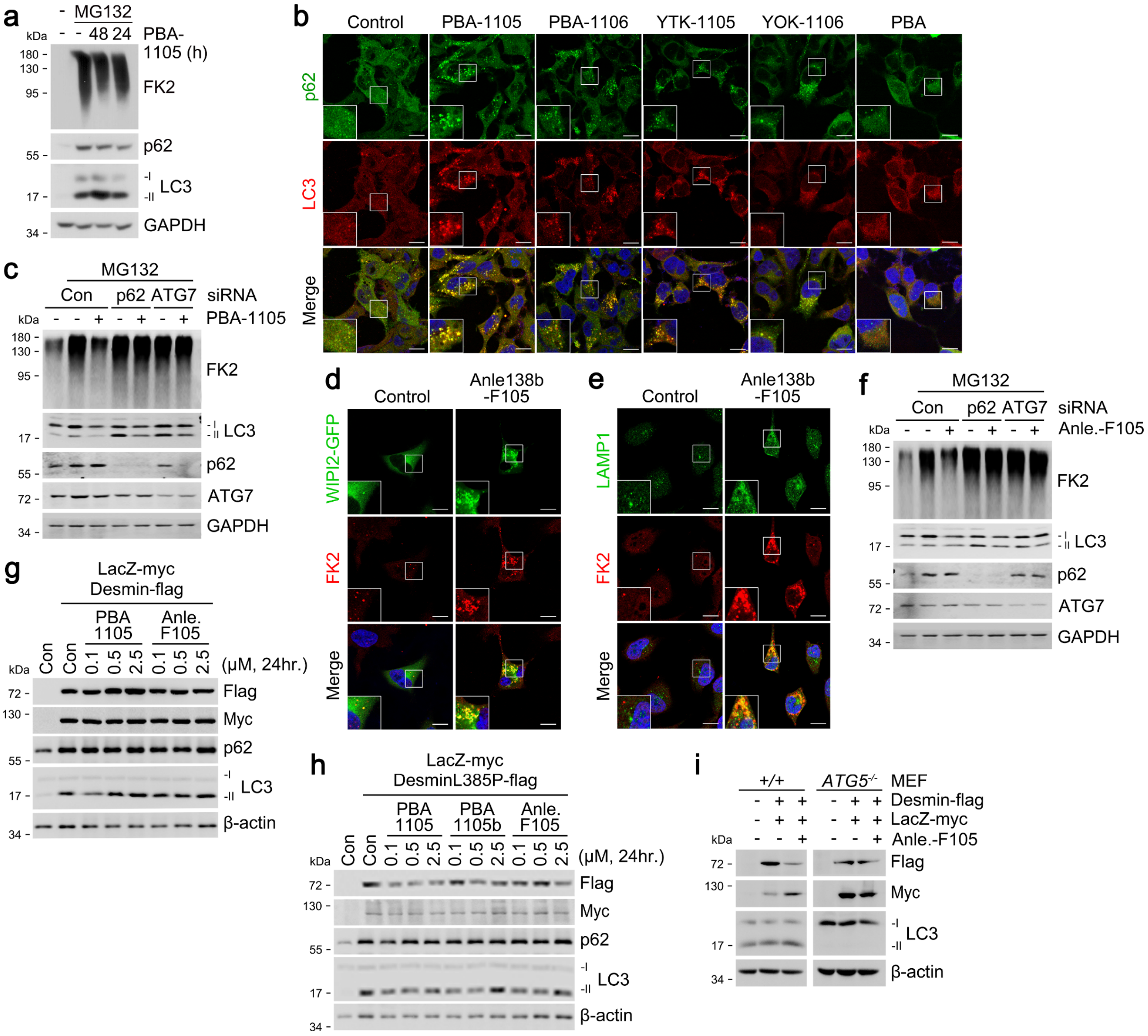


**Supplementary Fig. 5. AUTOTACs selectively target pathological aggregation-prone proteins for autophagic degradation. (a)** WB in HEK293T cells treated with PBA-1105 (1 μM) and MG132 (1 μM, 24 h) at the indicated time points. **(b)** ICC of HeLa cells treated with the indicated compounds (1 μM, 24 h). Scale bar, 10 μm. **(c)** WB in HEK293T cells treated with PBA-1105 (500 nM, 24 h) and MG132 (1 μM, 24 h) under siRNA-mediated knockdown of *p62* or *ATG7* (20 nM, 48 h). **(d)** ICC in HeLa cells transiently expressing WIPI2-GFP and treated with Anle138b-F105 (1 μM, 24 h). Scale bar, 10 μm. **(e)** ICC in HeLa cells treated with Anle138b-F105 (1 μM, 24 h). Scale bar, 10 μm. **(f)** Identical to **c**, but with Anle138b-F105 (500 nM, 24 h). **(g, h)** WB in HeLa cells transiently expressing wild type or mutant (L385P) desmin-flag with LacZ-myc and treated with PBA-1105, PBA-1105b or Anle138b-F105 at the

indicated concentrations (24 h). **(i)** WB in wild-type or *ATG5*$^{-/-}$ mouse embryonic fibroblasts transiently expressing mutant desminL385P with LacZ-myc and treated with Anle138b-F105 (500 nM, 24 h). Source data are provided with this paper.

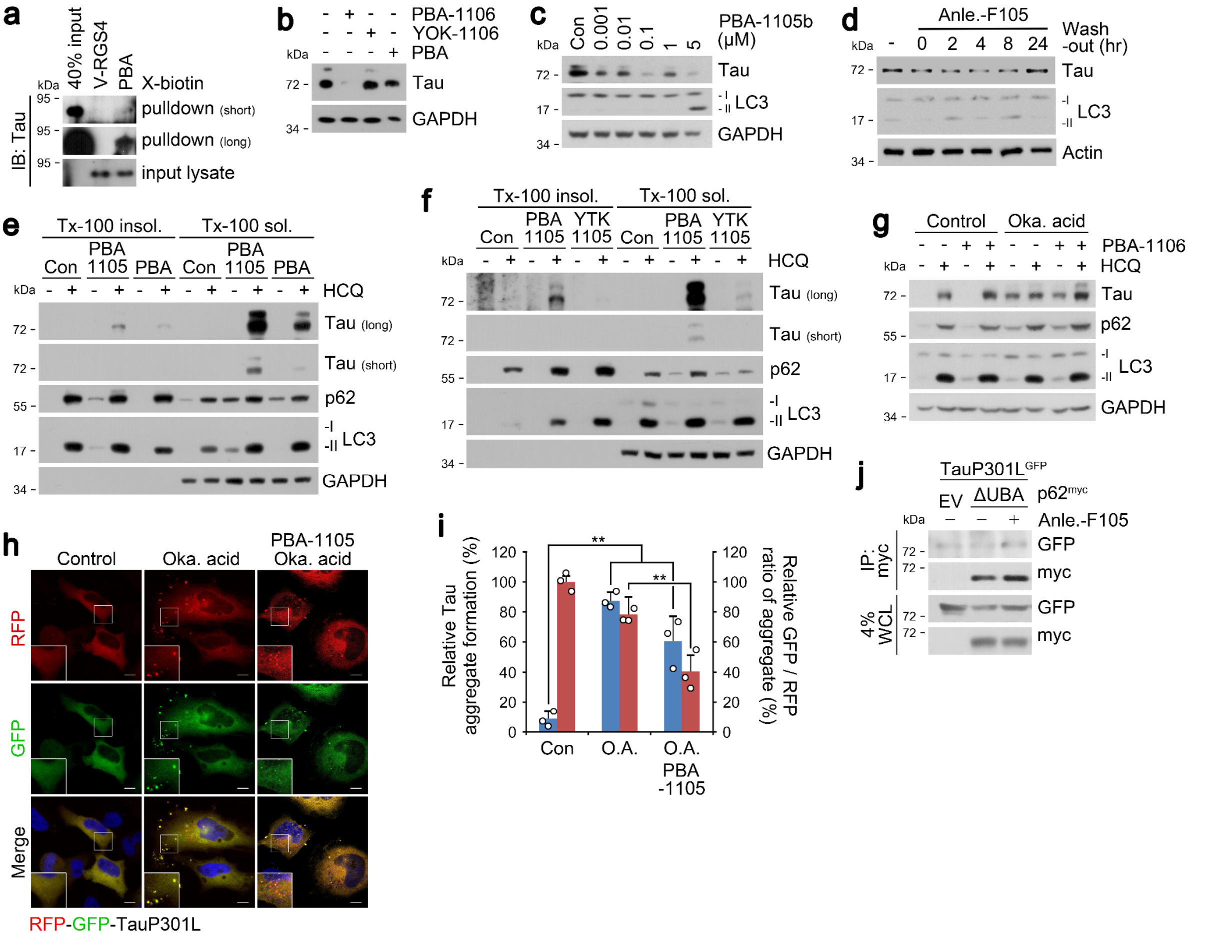


**Supplementary Fig. 6. AUTOTACs selectively degrade pathological aggregation-prone mutant tau. (a)** *In vitro* pull-down assay in HEK293T cells of biotinylated PBA, the N-terminal 12-mer V-BiP or V-RGS4 peptide. **(b)** WB in SH-SY5Y-tauP301L cells treated with PBA-1106, YOK-1106 or PBA (1 μM, 24 h). **(c)** WB in SH-SY5Y-tauP301L cells treated with PBA-1105b at the indicated concentrations (24 h). **(d)** WB in SY5Y-tauP301L cells treated with Anle138b-F105 (100 nM, 24 h) and washed-out for the indicated durations. **(e, f)** Triton X-100-insoluble fractionation assay in SH-SY5Y-tauP301L cells treated with PBA-1105 and PBA or YTK-1105 (2.5 μM, 24 h), respectively and HCQ (10 μM, 24 h). **(g)** WB in SH-SY5Y-tauP301L cells treated with HCQ (25 μM, 24 h), okadaic acid (15 nM, 24 h) and PBA-1106 (0.1 μM, 24 h). **(h)** ICC in HeLa cells transiently expressing mRFP-GFP-hTauP301L and treated with okadaic acid (15 nM, 24 h) prior to the presence or absence of PBA-1105 (100 nM,

24 h). Scale bar, 10 µm. **(i)** Quantification of **h** for punctate formation and GFP/RFP ratio (n=3 biologically independent experiments each counting 50 cells and 10 puncta within one image). Data are presented as mean values ± SD for aggregate formation and as mean values ± SEM for GFP/RFP ratio. **(j)** Co-IP assay in HEK293T cells co-transfected (24 h) with ΔUBA p62-myc and hTauP301L-GFP or negative control empty vector (EV), treated with Anle138b-F105 (1 µM, 24 h) or negative control. *P*-values (from a two-sided unpaired *t*-test): $^{**}P < 0.00662$ (for tau aggregate formation), $^{**}P < 0.00779$ (for GFP/RFP ratio). Source data are provided with this paper.

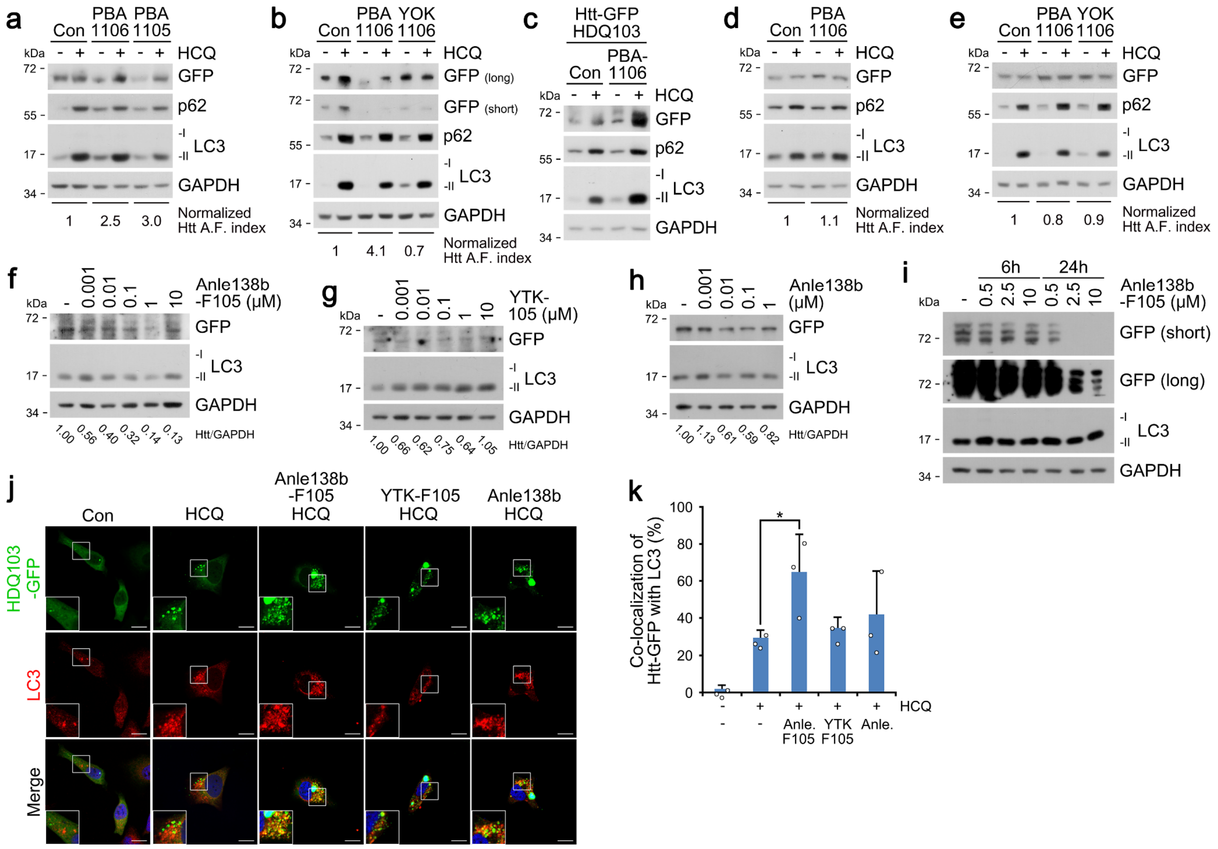

**Supplementary Fig. 7. AUTOTACs selectively degrade pathological mutant huntingtin.** **(a)** WB in HeLa-Htt-NLS-Q97-GFP cells treated with PBA-1106 or PBA-1105 (2.5 μM, 24 h) with or without HCQ (25 μM, 24 h). **(b)** Identical to **a** but with YOK-1106 instead of PBA-1105 in HeLa-Htt-NES-Q97-GFP cells. **(c)** WB in HeLa cells transfected with HDQ103-GFP and treated with PBA-1106 (2.5 μM, 24 h) and/or HCQ (25 μM, 24 h). **(d)** WB in HeLa-Htt-NLS-Q25-GFP cells treated with PBA-1106 (2.5 μM, 24 h) and/or HCQ (25 μM, 24 h). **(e)** Identical to **d**, but with PBA-1106 and YOK-1106 (2.5 μM, 24 h) in HeLa-Htt-NES-Q25-GFP cells. **(f, g, h, i)** WB in HeLa-Htt-NLS-Q97-GFP cells treated with Anle138b-F105, YTK-105 or Anle138b with the indicated concentrations and time points. **(j)** ICC of HeLa cells expressing HDQ103-GFP with the indicated compounds (1 μM, 24 h) treated with HCQ (10 μM, 24 h). Scale bar, 10 μm. **(k)** Quantification of **j** (n=3 biologically independent experiments each counting 50 cells). *P*-values (from a two-sided unpaired *t*-test): **P* < 0.0432. Data are presented as mean values ± SD where relevant. Source data are provided with this paper.

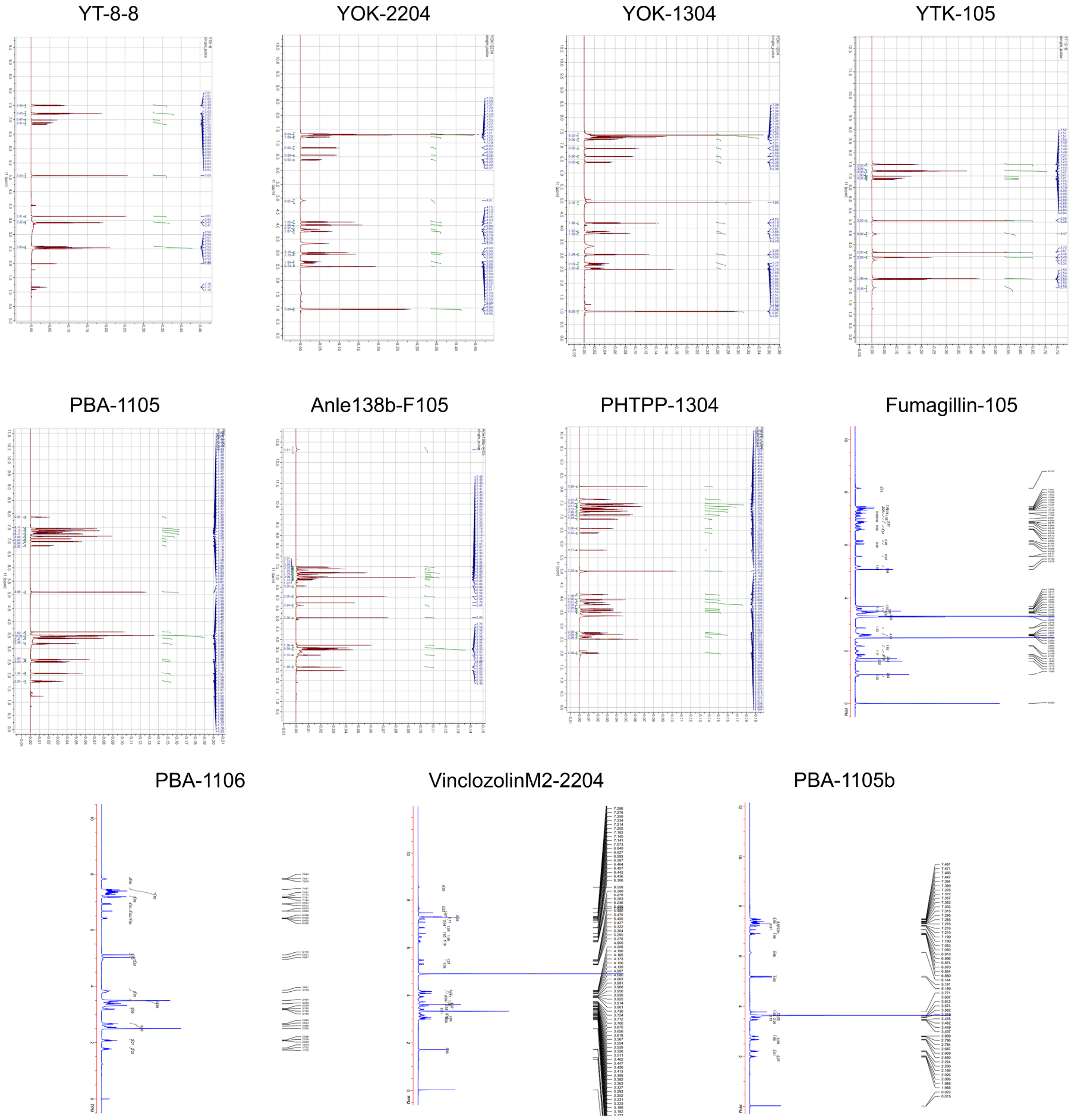


**Supplementary Fig. 8. $^1$H-NMR spectrum of ATLs and AUTOTACs used in this study.** 500 MHz $^1$H-NMR spectrum of YT-8-8, YOK-2204, YOK-1304, YTK-105, PBA-1105, Anle138b-F105, PHTPP-1304 in DMSO_$d_6$ and 400 MHz $^1$H-NMR spectrum of Fumagillin-105, PBA-1106, VinclozolinM2-2204 and PBA-1105b in DMSO_$d_6$.

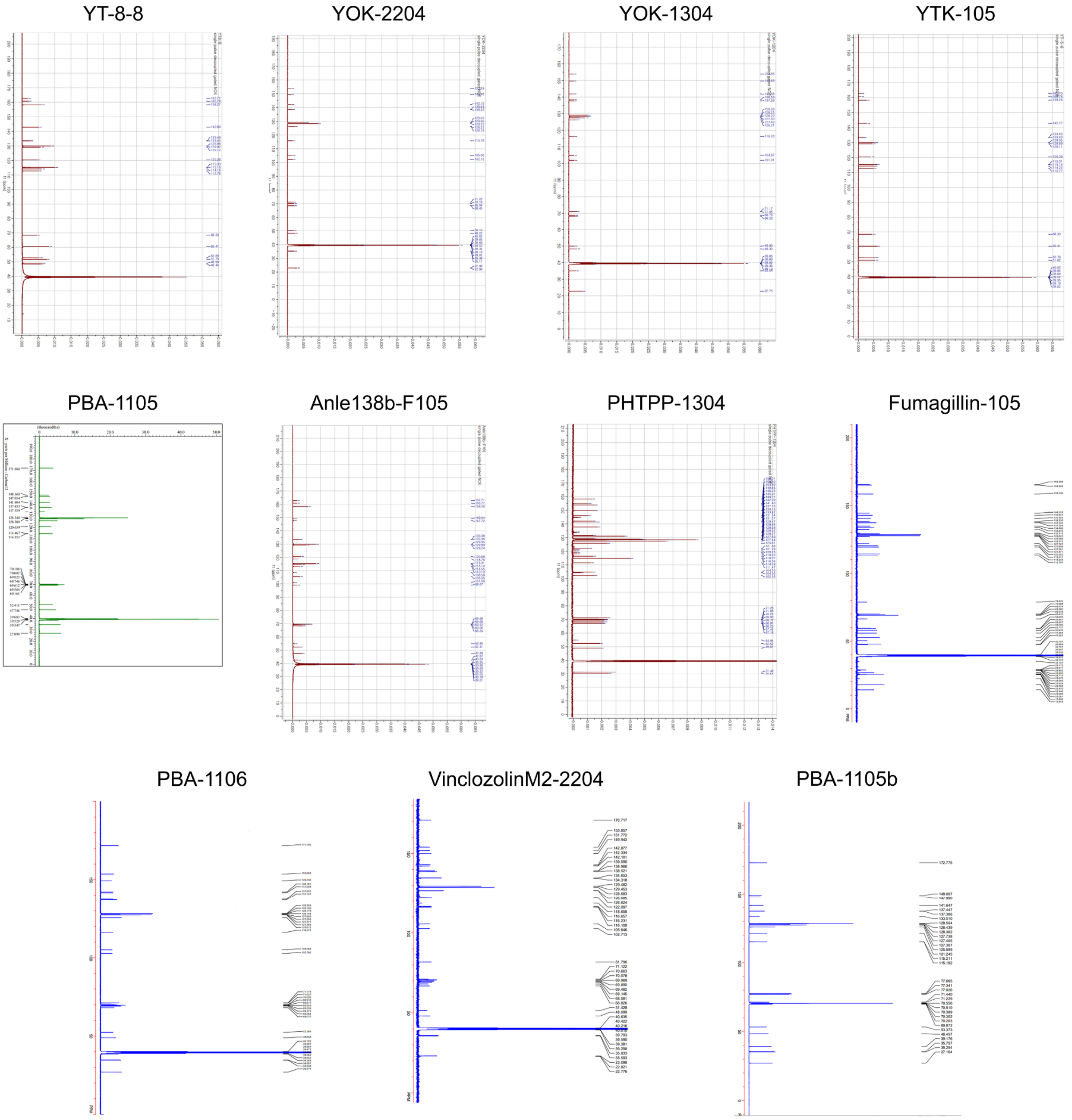


**Supplementary Fig. 9. $^{13}$C-NMR spectrum of ATLs and AUTOTACs used in this study.** 125 MHz $^{1}$H-NMR spectrum of YT-8-8, YOK-2204, YOK-1304, YTK-105, PBA-1105, Anle138b-F105, PHTPP-1304 in DMSO_$d_6$ and 100 MHz $^{1}$H-NMR spectrum of Fumagillin-105, PBA-1106, VinclozolinM2-2204 in DMSO_$d_6$ and PBA-1105b in $CDCl_3$.

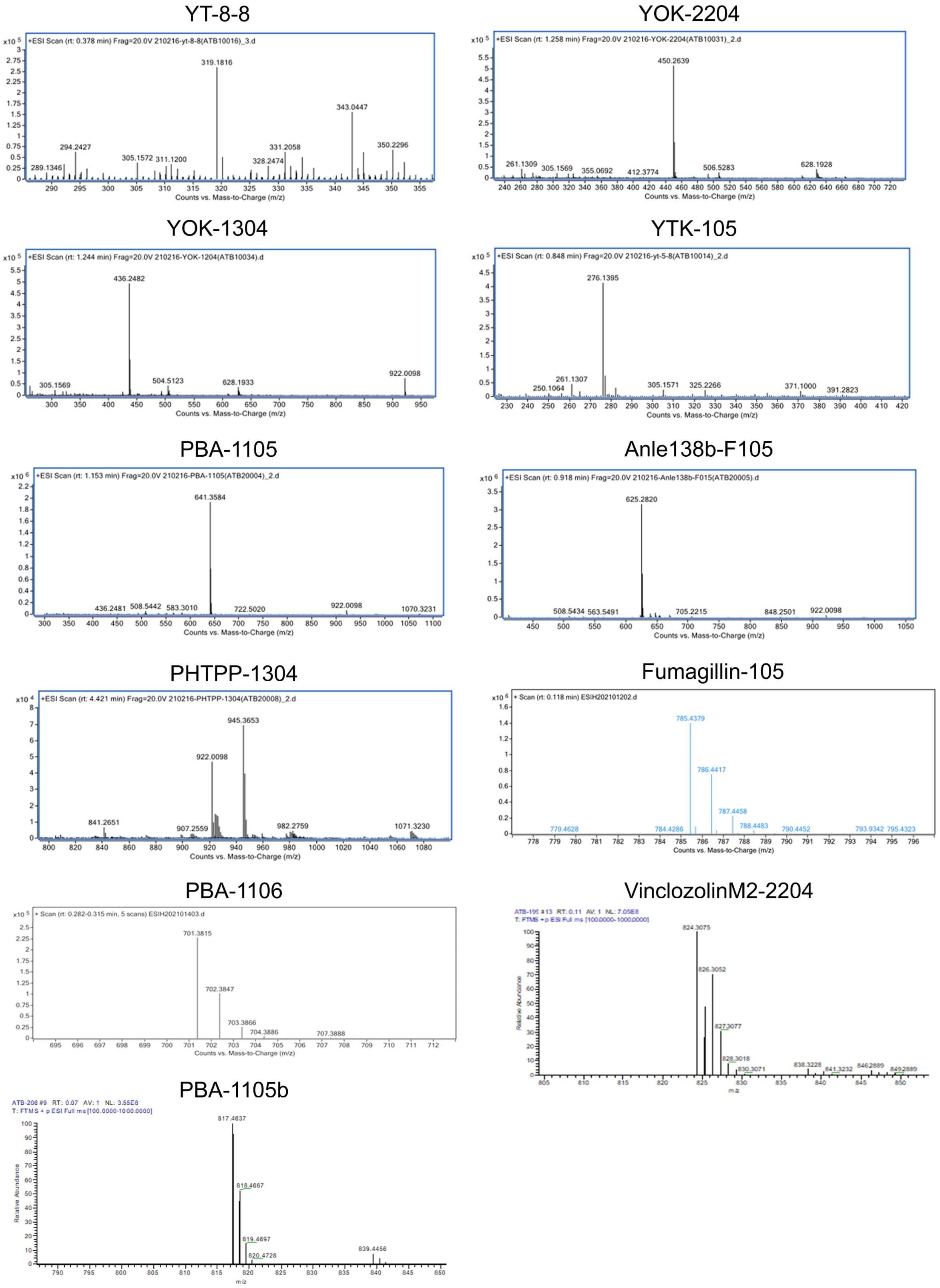


**Supplementary Fig. 10. HR-MS data of ATLs and AUTOTACs used in this study.** HRMS data of YT-8-8, YOK-2204, YOK-1304, YTK-105, PBA-1105, Anle138b-F105, PHTPP-1304, Fumagillin-105, PBA-1106, VinclozolinM2-2204, PBA-1105b.

## PK parameters of PBA-1105

| Route | IV | PO | IP |
|---|---|---|---|
| Dose / Unit (mpk) | 5 | 20 | 10 |
| $T_{1/2}$ (hr) | 1.53 | 1.15 | 1.17 |
| $T_{max}$ (hr) | | 0.42 | 0.50 |
| $C_{max}$ (ng/ml) | 886.0 | 124.7 | 703.4 |
| $AUC_{last}$ (h*hg/ml) | 1064.7 | 221.6 | 1012.2 |
| F (%) | | 5.2 | |

**Supplementary Table 1. PK parameters of PBA-1105.** PK parameters of PBA-1105 (single injection) in ICR mice.
