# Supplementary material for: The AUTOTAC chemical biology platform for targeted protein degradation via the autophagy-lysosome system
Source: Nat Commun. 2022 Feb 16;13:904. doi: 10.1038/s41467-022-28520-4 (PMC8850458; doi:10.1038/s41467-022-28520-4)
Supplement: Supplementary file 3 — Reporting Summary [file 41467_2022_28520_MOESM3_ESM.pdf]

nature research

Corresponding author(s): Yong Tae Kwon

Last updated by author(s): Dec 8, 2021

Reporting Summary

Nature Research wishes to improve the reproducibility of the work that we publish. This form provides structure for consistency and transparency in reporting. For further information on Nature Research policies, see our Editorial Policies and the Editorial Policy Checklist.

Statistics

For all statistical analyses, confirm that the following items are present in the figure legend, table legend, main text, or Methods section.

n/a	Confirmed	
☐	☒	The exact sample size (*n*) for each experimental group/condition, given as a discrete number and unit of measurement
☐	☒	A statement on whether measurements were taken from distinct samples or whether the same sample was measured repeatedly
☐	☒	The statistical test(s) used AND whether they are one- or two-sided<br>*Only common tests should be described solely by name; describe more complex techniques in the Methods section.*
☒	☐	A description of all covariates tested
☒	☐	A description of any assumptions or corrections, such as tests of normality and adjustment for multiple comparisons
☐	☒	A full description of the statistical parameters including central tendency (e.g. means) or other basic estimates (e.g. regression coefficient) AND variation (e.g. standard deviation) or associated estimates of uncertainty (e.g. confidence intervals)
☒	☐	For null hypothesis testing, the test statistic (e.g. *F*, *t*, *r*) with confidence intervals, effect sizes, degrees of freedom and *P* value noted<br>*Give P values as exact values whenever suitable.*
☒	☐	For Bayesian analysis, information on the choice of priors and Markov chain Monte Carlo settings
☒	☐	For hierarchical and complex designs, identification of the appropriate level for tests and full reporting of outcomes
☒	☐	Estimates of effect sizes (e.g. Cohen's *d*, Pearson's *r*), indicating how they were calculated

Our web collection on statistics for biologists contains articles on many of the points above.

Software and code

Policy information about availability of computer code

Data collection	Immunocytochemistry images were captured using Zeiss Zen Blue edition (version 3.4)<br>Fluorescence and total area of each puncta in immunocytochemistry assays were measured using ImageJ (version 1.53n 7; NIH, Bethesda).<br>Densitometry of Western blot bands was measured and analyzed with ImageJ (version 1.53n 7; NIH, Bethesda).<br>Flow cytometry was measured and analyzed with Modfit LT (version 5.0; Verity Software House).
Data analysis	Immunocytochemistry images were analyzed using Zeiss Zen Blue edition (version 3.4)<br>P-values were determined using unpaired, two-tailed student's t-test (degree of freedom = n-1) with Prism 6 software (version 6.07; GraphPad).<br>Fluorescence and total area of each puncta in immunocytochemistry assays were measured using ImageJ (version 1.53n 7; NIH, Bethesda).<br>Densitometry of Western blot bands was measured and analyzed with ImageJ (version 1.53n 7; NIH, Bethesda).<br>Flow cytometry was measured and analyzed with Modfit LT (version 5.0; Verity Software House).

For manuscripts utilizing custom algorithms or software that are central to the research but not yet described in published literature, software must be made available to editors and reviewers. We strongly encourage code deposition in a community repository (e.g. GitHub). See the Nature Research guidelines for submitting code & software for further information.

Data

Policy information about availability of data

All manuscripts must include a data availability statement. This statement should provide the following information, where applicable:

- Accession codes, unique identifiers, or web links for publicly available datasets
- A list of figures that have associated raw data
- A description of any restrictions on data availability

All data generated in this study are provided in the article and its associated files, including the Supplementary Information and Source Data files, which are provided with this paper.

Field-specific reporting

Please select the one below that is the best fit for your research. If you are not sure, read the appropriate sections before making your selection.

☒ Life sciences ☐ Behavioural & social sciences ☐ Ecological, evolutionary & environmental sciences

For a reference copy of the document with all sections, see nature.com/documents/nr-reporting-summary-flat.pdf

Life sciences study design

All studies must disclose on these points even when the disclosure is negative.

Sample size	Replicates among experiments were both biologically and technically independent, carried out at different time points and different persons (where possible) to maximize reproducibility.<br>For immunocytochemistry assays and puncta counting analyses, 50 cells were counted three times.<br>For immunoblotting assays and densitometry analyses, three independent experiments were performed and analyzed.<br>For wound healing assays, an average diameter of the wound consisting of three random measurements was duplicated or triplicated.<br>For WST-based cell viability assays, two biologically independent measurements were taken.<br>Mice experiments were performed two independent times (n=4 and n=7, respectively), with only the latter presented in this study.<br>Where possible, sample sizes were chosen based on established protocols in previous publications and/or generally-accepted criteria in the scientific community.
Data exclusions	No data were excluded from the analyses.
Replication	Replicates among experiments were both biologically and technically independent, carried out at different time points and different persons (where possible) to maximize reproducibility.<br>For immunocytochemistry assays and puncta counting analyses, 50 cells were counted three times.<br>For immunoblotting assays and densitometry analyses, three independent experiments were performed and analyzed.<br>For wound healing assays, an average diameter of the wound consisting of three random measurements was duplicated or triplicated.<br>For WST-based cell viability assays, two biologically independent measurements were taken.<br>Mice experiments were performed two independent times (n=4 and n=7, respectively), with only the latter presented in this study.<br>All in vitro and in vivo experiments were replicated succesfully.
Randomization	All samples, cells and animals were randomized in this study.
Blinding	Investigators were blinded to group allocation during data collection and analysis.

Reporting for specific materials, systems and methods

We require information from authors about some types of materials, experimental systems and methods used in many studies. Here, indicate whether each material, system or method listed is relevant to your study. If you are not sure if a list item applies to your research, read the appropriate section before selecting a response.

Materials & experimental systems

n/a	Involved in the study	
☐	☒	Antibodies
☐	☒	Eukaryotic cell lines
☒	☐	Palaeontology and archaeology
☐	☒	Animals and other organisms
☒	☐	Human research participants
☒	☐	Clinical data
☒	☐	Dual use research of concern

Methods

n/a	Involved in the study	
☒	☐	ChIP-seq
☐	☒	Flow cytometry
☒	☐	MRI-based neuroimaging

Antibodies

Antibodies used	The antibodies used in this study are as follows: mouse monoclonal anti-p62 (Abcam, ab56416, 1:10,000), rabbit polyclonal anti-LC3 (Sigma, L7543, 1:10,000), rabbit polyclonal anti-ATE1 (Sigma, HPA038444, 1:1000), mouse monoclonal anti-FK2 specific to Ub-conjugated proteins (Enzo, BML-PW8810, 1:3000), rabbit polyclonal anti-GAPDH (BioWorld, AP0063, 1:20,000), rabbit polyclonal anti-b-actin (BioWorld, AP0060, 1:20,000), mouse monoclonal anti-MetAP2 (Santa Cruz, sc-365637, 1:2000), rabbit polyclonal anti-ERβ (Invitrogen, PA1-310B, 1:2000), rabbit polyclonal anti-Androgen Receptor (Cell Signaling, 3202, 1:5000), rabbit polyclonal anti-EGFR (Cell Signaling, 4265, 1:2000), rabbit polyclonal anti-p-Akt (Cell Signaling, 9271, 1:2000), rabbit polyclonal anti-Akt (Cell Signaling, 2920, 1:1000), rabbit polyclonal anti-p-ERK (Cell Signaling, 9101, 1;1000), rabbit polyclonal anti-ERK (Cell Signaling, 9102, 1:1000), rabbit polyclonal anti-ATG5 (Novus, NB110-53818, 1:1000), mouse monoclonal anti-Ub (Santa Cruz, sc-8017, 1:2000), mouse monoclonal anti-GFP (Santa Cruz, sc-9996, 1:2000), mouse monoclonal anti-Tau5 (Invitrogen, AHB0042, 1:5000), rabbit polyclonal anti-p-Tau (Invitrogen, 44-752G, 1:5000). The following secondary antibodies were used: alexa fluor 488 goat anti-rabbit IgG (Invitrogen, A11034, 1:1000), alexa fluor 488 goat anti-mouse IgG (Invitrogen, A11029, 1:1000), alexa fluor 555 goat anti-rabbit IgG (Invitrogen, A32732, 1:1000), alexa fluor 555 goat anti-mouse IgG (Invitrogen, A32727, 1:1000), anti-rabbit IgG-HRP (Cell Signaling, 7074, 1:10,000), and anti-mouse IgG-HRP (Cell Signaling, 7076, 1:10,000).
Validation	Each primary antibody was validated according to the manufacturer's website and by immunoblotting in this study.<br>Specifically, Abcam antibodies were validated by knock-out, Sigma antibodies by functional assays detecting experimentally induced changes in target antigen expression, BioWorld antibodies by functional immunoblotting, Santa Cruz antibodies by functinoal immunoblotting, immunocytochemistry and ELISA, Invitrogen antibodies by functional cell treatment, and Cell Signaling antibodies by functional assays, as well as knock-out studies, detecting experimentally induced changes in target antigen expression.

Eukaryotic cell lines

Policy information about cell lines

Cell line source(s)	HeLa, HEK293, HEK293T, U-87 MG, ACHN, MCF7 and LNCaP cell lines were obtained from ATCC. SH-SY5Y-tauP301L-GFP was obtained from Innoprot (P30722). HeLa-NLS/NES-Htt-Q25/Q97-eGFP cell lines were a gift from Min Jae Lee (originally created by Min Jae Lee's lab at Seoul National University, Korea)
Authentication	Every stable cell line was authenticated by detection of target protein via immunoblotting and immunocytochemistry.
Mycoplasma contamination	All cell lines tested negative for mycoplasma contamination.
Commonly misidentified lines (See ICLAC register)	None.

Animals and other organisms

Policy information about studies involving animals; ARRIVE guidelines recommended for reporting animal research

Laboratory animals	Both males and females of 9-month-old hTau-P301L- BiFC mice (C57BL/6 strain) were used.<br>Male ICR mice (6 months old; ICR/HaJ strain) were used.<br>Mice were bred and housed in a 12:12 light-dark cycle, pathogen-free, temperature- and humidity-controlled facility with food and water available at Korea Institute of Science and Technology. Animal protocols followed the principles and practices outlined in the approved guidelines by the Institutional Animal Care and Use Committee of the Korea Institute of Science and Technology.
Wild animals	The study did not involve wild animals.
Field-collected samples	The study did not involve samples collected from the field.
Ethics oversight	Animal protocols followed the principles and practices outlined in the approved guidelines by the Institutional Animal Care and Use Committee of the Korea Institute of Science and Technology.

Note that full information on the approval of the study protocol must also be provided in the manuscript.

Flow Cytometry

Plots

Confirm that:

- [x] The axis labels state the marker and fluorochrome used (e.g. CD4-FITC).
- [x] The axis scales are clearly visible. Include numbers along axes only for bottom left plot of group (a 'group' is an analysis of identical markers).
- [x] All plots are contour plots with outliers or pseudocolor plots.
- [x] A numerical value for number of cells or percentage (with statistics) is provided.

Methodology

Sample preparation	Cell death and cell cycle arrest were quantified by staining cells with propidium iodide for flow cytometry with a BD

	FACSCalibur (BD Biosciences) according to the manufacturer's instructions. Briefly, 1x106 cells were incubated with fumagillin-105 (1 µM, 48 h) or negative control DMSO. Cells were collected by centrifugation and fixed in 70% ethanol at 4°C for 24 h. Cells were washed with PBS and stained with propidium iodide (10 µg/mL) with RNAse treatment at 37°C for 30 min. DNA content at each cell cycle checkpoint was measured and analyzed using BD CellQuest Pro (BD Biosciences) and ModFit LT Systems (Verity Software House).
Instrument	BD LSR II System (BD Biosciences)
Software	BD CellQuest Pro software and ModFit LT analysis (Verity Software House).
Cell population abundance	Of the 900+ cells counted and sorted, over 850 (>94%) exceeded 90% purity, and over 423 (47%) exceeded 99% purity.
Gating strategy	Cells were stained with propidium iodide. Since the FACS data in question only analyzed rudimentary cell cycle phases without additional markers specific to live cells or dead cells, all cells were visualized and counted. Doublets were eliminated and only singlets were counted.

☒ Tick this box to confirm that a figure exemplifying the gating strategy is provided in the Supplementary Information.
